# Oligomerization regulates the interaction of Gemin5 with members of the SMN complex and the translation machinery

**DOI:** 10.1038/s41420-024-02057-5

**Published:** 2024-06-28

**Authors:** Rosario Francisco-Velilla, Salvador Abellan, Azman Embarc-Buh, Encarnacion Martinez-Salas

**Affiliations:** Centro de Biología Molecular Severo Ochoa, CSIC-UAM, Nicolás Cabrera 1, 28049 Madrid, Spain

**Keywords:** Molecular biology, Cell biology

## Abstract

RNA-binding proteins are multifunctional molecules impacting on multiple steps of gene regulation. Gemin5 was initially identified as a member of the survival of motor neurons (SMN) complex. The protein is organized in structural and functional domains, including a WD40 repeats domain at the N-terminal region, a tetratricopeptide repeat (TPR) dimerization module at the central region, and a non-canonical RNA-binding site at the C-terminal end. The TPR module allows the recruitment of the endogenous Gemin5 protein in living cells and the assembly of a dimer in vitro. However, the biological relevance of Gemin5 oligomerization is not known. Here we interrogated the Gemin5 interactome focusing on oligomerization-dependent or independent regions. We show that the interactors associated with oligomerization-proficient domains were primarily annotated to ribosome, splicing, translation regulation, SMN complex, and RNA stability. The presence of distinct Gemin5 protein regions in polysomes highlighted differences in translation regulation based on their oligomerization capacity. Furthermore, the association with native ribosomes and negative regulation of translation was strictly dependent on both the WD40 repeats domain and the TPR dimerization moiety, while binding with the majority of the interacting proteins, including SMN, Gemin2, and Gemin4, was determined by the dimerization module. The loss of oligomerization did not perturb the predominant cytoplasmic localization of Gemin5, reinforcing the cytoplasmic functions of this essential protein. Our work highlights a distinctive role of the Gemin5 domains for its functions in the interaction with members of the SMN complex, ribosome association, and RBP interactome.

## Introduction

RNA-binding proteins (RBPs) are a diverse group of ubiquitous factors assisting fundamental cellular processes, from genome transcription to splicing, RNA stability, and translation [1–3]. A prominent feature of RBPs is their modular organization in structural and functional RNA-binding domains (RBDs), which determines the interaction with their partners [4–7]. Noteworthy, the implementation of novel methodologies to capture RBPs evidenced that many RBPs lack conventional RBDs and also that the number of RBPs in human cells is greater than anticipated [8, 9].

Despite the knowledge accumulated on a limited number of specific RBPs [10, 11], a systematic functional study of the vast majority of RBPs remains elusive [12]. The results obtained from global genomic and proteomic investigations support the view that RBPs play key roles in sustaining cell proliferation in living organisms, such that the expression of variant proteins is associated with disease. This view reinforces the importance of a comprehensive analysis of understudied RBPs to enhance our understanding of their implication in health and disease.

Gemin5 was first identified as the RBP of the survival of motor neurons (SMN) complex [13, 14]. This macromolecular complex consists of eight core proteins (SMN, Gem-associated (Gemins2–8), and unr-interacting protein (Unrip)) in higher eukaryotes [15–18]. Gemin5 was reported to recognize the U-rich sequence of small nuclear RNAs (snRNAs) and deliver to small nuclear ribonucleoproteins (snRNPs) [19]. In addition, Gemin5 has been associated to various processes impacting on gene expression, including gene reprogramming and translation regulation [20–26]. This versatile function is in line with the observation that a large fraction of Gemin5 is found in the cytoplasm outside of the SMN complex [27].

Gemin5 has been shown to be essential in fly and mouse models, as KO mutant genes are embryonic lethal [28, 29]. In further support of its critical role for cell growth and survival, biallelic variants were recently identified in patients developing neurological disorders [30–34]. Nevertheless, as it occurs with many other understudied proteins, the molecular basis of Gemin5 dysfunction remains poorly understood. Dimerization defects in Gemin5 variants described in patients with neurological disorders resulted in the loss of interaction with factors connected to RNA processing, translation regulation, and spliceosome assembly [32], establishing a link between the pathogenic mutations and protein malfunction. This observation led us to hypothesize that a critical feature of the physiological role of Gemin5 relies on providing a hub for the assembly of macromolecular complexes.

**Fig. 1:**
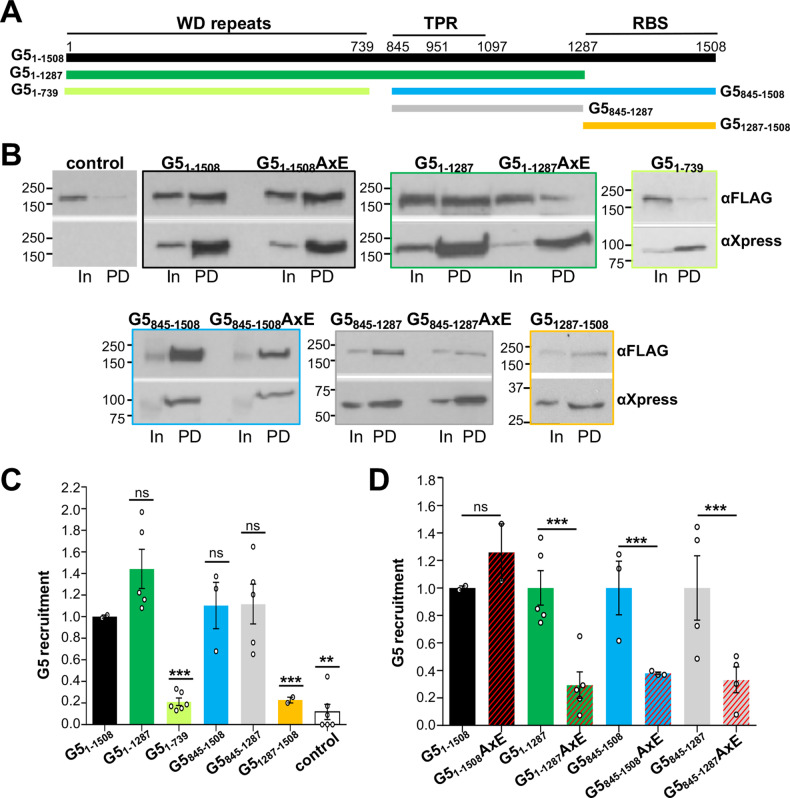
The TPR module is essential to form Gemin5 oligomers in living cells. **A** Schematic of Gemin5 domains. The regions including the WD40 repeats, the TPR dimerization module, and the non-canonical RNA-binding site are plotted. The position of amino acids flanking each domain and the dimerization mutation (A951E, then AxE) are indicated at the top. The different Gemin5 proteins used in the assay are denoted by color bars. The amino acids encompassing each protein are indicated. **B** HEK293 cells expressing G5_1–1508_-FLAG and the corresponding Xpress-His construct (control, G5_1–1508_, G5_1–1508_AxE, G5_1–1287_, G5_1–1287_AxE, G5_1–739_, G5_845–1508_, G5_845–1508_AxE, G5_845–1287_, G5_845–1287_AxE, or G5_1287–1508_) were lysed and subjected to pull-down assays. Protein expression (In, Inputs) and pull-down (PD) samples of independent experiments (*n* = 2 for G5_1–1508_, G5_1–1508_AxE, G5_1287–1508_; *n* = 3 for G5_845–1508_, G5_845–1508_AxE; *n* = 5 for G5_1–1287_, G5_1–1287_AxE, G5_845–1287_; *n* = 6 for control, G5_1–739_) were analyzed by western blot (WB) using anti-FLAG and anti-Xpress antibodies. Histograms depicting Gemin5 (G5) recruitment measured by relative intensity (PD/In) of G5_1–1508_-FLAG recruited with the Xpress-His wild-type proteins (**C**), or the Xpress-His A951E mutants (**D**). In all cases, the intensity of the bands was normalized by the PD/In ratio obtained in the control cells performed side by side. Values represent the mean ± SEM, asterisks denote *P* values (ns not significant, **P < 0.01, ***P < 0.001).

The Gemin5 protein (1508 amino acids) comprises a singular combination of structural and functional domains not commonly found side by side in RBPs which determines its involvement in a broad range of cellular processes (Fig. 1A). The N-terminal half (G5_1–739_) contains two juxtaposed seven-bladed WD40 repeats domains (named WD1 and WD2) [35] forming a contiguous RNA-binding surface that recognize the Sm site of snRNAs and the cap structure via base-specific interactions [35–37]. The C-terminal half (G5_845–1508_) encloses two different domains, a dimerization module in the central region [38], and a non-canonical RNA-binding site at the most C-terminal end (G5_1287–1508_) [39]. Structural analysis of the central region identified a tetratricopeptide repeat (TPR) domain with 17 α-helices that dimerize as a canoe-shaped homodimer [38]. The most C-terminal part folds into a pentamer structure [40] such that the G5_842–1508_ region adopts a decamer architecture arranged as a dimer of pentamers. This structural conformation determines the RNA-binding and translation regulation properties of the C-terminal half of the protein, in accordance with previous results [41].

To determine whether the oligomerization capacity of Gemin5 regulates the activity of the protein, we performed a systematic biochemical analysis. Then, we investigated its interactome along with its functional properties and subcellular localization by using oligomerization-deficient mutants and truncated regions of the protein that differ in the oligomerization capacity. We found that distinct functions of Gemin5, concerning interaction with members of the SMN complex and the ribosomal machinery, depend upon the presence of a fully active oligomerization domain. Accordingly, mutants defective on the dimerization module, as well as truncated proteins lacking the TPR domain, fail to interact with factors belonging to these macromolecular complexes. However, Gemin5 association with polysomes also requires the WD40-repeat domain, indicating a major difference in the requirement for its association with distinct macromolecular complexes.

## Results

### Gemin5 recruitment requires an active TPR domain

To determine the contribution of the Gemin5 domains to protein recruitment in the cellular context, we tested the ability of the individual domains to form a stable complex with Gemin5. To this end, the full-length protein (G5_1–1508_) fused to a FLAG tag at the C-terminus, and different versions of Xpress-His tagged proteins (G5_1–739_, G5_1–1287_, G5_845–1508_, G5_845–1287_, and G5_1287–1508_) encompassing the WD40 repeats, the TPR, and the RBS domains [38, 39] were individually expressed in HEK293 cells (Fig. 1A). Expression of all proteins was verified by western blot in Input samples (In) (Fig. 1B). Pull-down (PD) of the FLAG-tagged Gemin5 protein was analyzed to verify the capacity of the expressed Xpress-His proteins to recruit Gemin5. Validating the specificity of this assay, a negative result was observed in the control sample (Fig. 1B). In contrast, recruitment of the Gemin5-FLAG was observed in all the Xpress-His-proteins that harbor the TPR domain such as G5_1–1287_, G5_845–1508_, and G5_845–1287_, but not in Xpress-His-proteins without TPR, as G5_1–739_ and G5_1287–1508_ (Fig. 1B, C).

Reinforcing these results, the analysis of endogenous Gemin5 recruitment measured by LC–MS/MS analysis as the ratio of peptide reads (740–1508/1–739 for G5_1–739_ relative to 1–844/845–1508 for G5_845–1508_) (Supplementary Fig. S1) revealed the strict requirement of the intact dimerization module for Gemin5 protein coprecipitation, as observed in G5_845–1508_ but not in G5_1–739_ (Supplementary Fig. S1). These results indicate that only proteins carrying the TPR domain elicit the capacity to recruit the Gemin5 protein, presumably forming an oligomer.

To confirm that the intact dimerization module was responsible for this property, we made use of the A951E (AxE) substitution, previously shown to abolish TPR dimerization [38]. The presence of this mutation in the TPR domain of proteins G5_1–1287_, G5_845–1508_, and G5_845–1287_ impaired Gemin5 recruitment (Fig. 1B, D). Strikingly, when the A951E mutation was placed in the Xpress-His protein (G5_1–1508_AxE) there was no decrease in the recruitment of the endogenous protein, suggesting the existence of additional contacts that either contribute or stabilize the complex in living cells.

Next, we investigated the presence of protein complexes resistant to extra amounts of DTT in cellular lysates expressing the individual domains of Gemin5 (Supplementary Fig. S2). The results showed that the Gemin5 proteins comprising an intact TPR domain (G5_1–1508_, G5_1–1287_, G5_845–1508_, G5_845–1287_) assemble oligomers, detectable in the presence of additional amount of DTT. In contrast, Xpress-tagged Gemin5 proteins lacking the TPR module (G5_1–739_) or containing the mutation A951E (G5_1–1287_AxE, G5_845–1508_AxE, G5_845–1287_AxE), do not have the ability to form oligomers under similar conditions. The full-length protein with the mutation A951E (G5_1–1508_AxE) forms stable oligomers according to the results shown in Fig. 1B, D.

We conclude that Gemin5 oligomerization depends on the presence of an active TPR module, although additional contacts between other regions of the protein, WD40, and RBS domains, contribute as well to Gemin5 oligomerization.

### The RNA-binding ability of Gemin5 contributes to protein oligomerization

The C-terminal half of Gemin5 (G5_842–1508_) folds as a dimer of pentamers, adopting a three-dimensional structure which is required for binding to its cognate RNA targets [40]. However, given that the TPR moiety (G5_845–1097_) failed to bind RNA using various probes that differ in sequence and secondary structure [38] we asked whether the TPR-extended region (G5_845–1287_) could form complexes with RNA. RNA-binding assays performed with increasing concentrations of purified His-G5_845–1287_ revealed that this version of the protein interacts with both d5 and SL1 RNA targets (Supplementary Fig. S3A, B), alike the His-RBS1 protein (G5_1287–1412_), previously shown to bind each of these RNAs [40, 41]. This result indicates that the ability of Gemin5 to recognize RNA depends on multiple residues scattered along the C-terminal half of the protein.

The presence of an extended RNA-binding surface [40, 41] on the C-terminal half of the protein together with a robust dimerization module [38], responsible for the recruitment of Gemin5 (Fig. 1), prompted us to investigate whether RNA-binding was contributing to Gemin5 oligomerization. To address this question, we analyzed by mass spectrometry the total reads of the peptides corresponding to the N-terminus (residues 1–845) of Gemin5 recruited with G5_845–1508_ with and without RNase A, as an estimation of the amount of endogenous interacting Gemin5 through RNA bridges. The RNase A treatment significantly reduced the ratio of peptide reads relative to the untreated G5_845–1508_ (Supplementary Fig. S3C), suggesting that RNA bridges stabilize Gemin5 oligomers.

### The oligomerization state of Gemin5 determines the protein interactome

Next, we sought to investigate the role of protein oligomerization on the Gemin5 interactome. To this end, we expressed and purified TAP-tagged proteins in human cells corresponding to both, the oligomerizing regions (G5_1–1287_ and G5_845–1508_) and those that do not oligomerize (G5_1–739_ and G5_845–1508_AxE) to identify the interacting partners by LC–MS/MS. For this qualitative study, we considered the overlap of factors detected in both biological replicates with >2 unique peptides (352 proteins for G5_1–1287_, 261 for G5_845–1508_, 169 for G5_1–739_, and 192 for G5_845–1508_AxE) (Dataset).

**Fig. 2:**
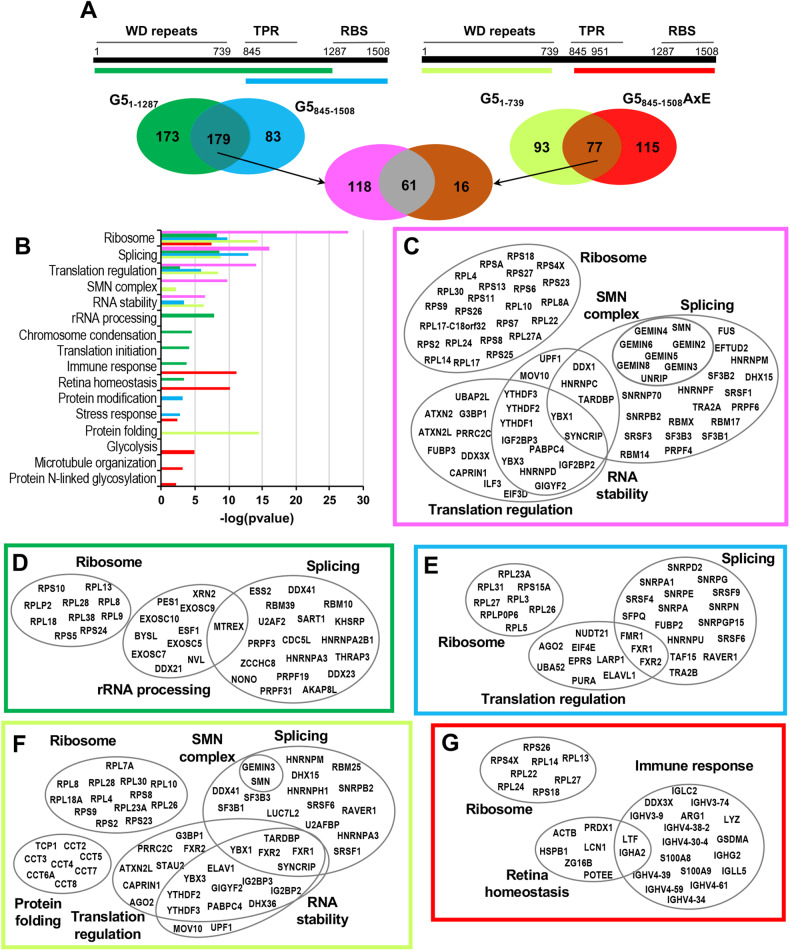
Networks associated with oligomerizing and oligomerization-deficient Gemin5 domains. **A** Schematic illustrating the flowchart followed to select the interactors considered in the interactome analysis. Venn diagrams depict the number of the overlapping factors in each case, colored as in the schematic protein regions. **B** Bar chart representing the gene ontology (GO) categories obtained for the overlapping and exclusive proteins, colored as in (**A**). Image depicting the name of proteins included in the GO groups (−log (*p* value) >5) for the shared factors (118) between G5_1–1287_ and G5_845–1508_ (**C**), factors preferentially interacting with G5_1–1287_ (173) (**D**), factors preferentially interacting with G5_845–1508_ (83) (**E**), factors preferentially associated with G5_1–739_ (93) (**F**), and **G** factors preferentially bound with G5_845–1508_AxE (115).

First, to define the interactome of Gemin5 we analyzed the shared and exclusive overlapping factors associated with oligomerization-proficient or deficient regions of Gemin5 (Fig. 2A). The oligomerizing regions shared 179 factors, while the oligomerization-deficient regions shared only 77 proteins. Since 61 of the latter group were in common with the oligomer-proficient regions of Gemin5 regardless of the oligomerization capacity, we reasoned that the unshared 118 factors were favored by protein oligomerization (Fig. 2A). Gene ontology (GO) analysis of the networks in the group of proteins associated to each of the overlapping and exclusive regions of the protein revealed main differences (Fig. 2B). GO classification of the 118 factors preferentially interacting with both oligomerization-dependent regions (G5_1–1287_ and G5_845–1508_) underscored five functional categories such as Ribosome, Splicing, Translation regulation, SMN complex, and RNA stability (Fig. 2B). Representative proteins of each of these GO classes are shown in Fig. 2C. Worth noting is the presence of the whole SMN complex, including SMN, Gemin-2, -3, -4, -5, -6, -8, and Unrip. In addition, several ribosomal proteins and translation regulatory factors were shared by all the oligomerization-proficient regions, strongly suggesting that Gemin5 oligomerization mediates ribosome binding and translation regulation. Curiously, the oligomerizing extended N-terminal region G5_1–1287_ displayed preferential interaction with rRNA processing, comprising 11 factors, including 4 members of the exosome complex (Fig. 2B, D), while the oligomerizing half C-terminal region G5_845–1508_ associated factors belonging to Ribosome, Translation regulation, and Splicing (Fig. 2B, E). Notably, the splicing network included 7 SNRP proteins suggesting a preferential binding to this oligomer conformation.

Despite retaining the ribosome network, a marked difference was observed in networks associated with the regions of the protein not involved in oligomerization, G5_1–739_ and G5_845–1508_AxE (Fig. 2B). Thus, major changes were observed in the network preferentially interacting with the WD40-repeat moiety (G5_1–739_), which included protein folding, comprising 8 members of the T-complex protein ring complex (TRIC complex) (Fig. 2F). We noticed that GO categories displayed by G5_1–739_ (Splicing, RNA stability, Translation regulation, and SMN complex) present fewer number of proteins and lower statistical significance than oligomerizing regions (Fig. 2B). In turn, prominent changes were observed in the interactome associated with the dimerization defective mutant G5_845–1508_AxE. This protein version recruited factors belonging to immune response and retina homeostasis (Fig. 2G), and it had lost the ability to interact with the vast majority of factors associated to both G5_1–739_ and G5_845–1508_. These results indicate that G5_1–739_ partially retains the capacity to form RNP complexes though to a lesser extent than the oligomerizing regions, whereas G5_845–1508_AxE loses the ability to interact with RBPs.

Second, a statistical analysis (*Z* score > 1.96, 95% confidence) of the factors associated to each of the Gemin5 regions based on the average peptide-spectrum match (AvePSM) revealed a differential specific partnership of the oligomerization-proficient proteins (Supplementary Table S1). Thus, factors identified with high *Z* score using G5_845–1508_ were lost in both oligomerization-deficient regions, G5_1–739_ and G5_845–1508_AxE. Interestingly, the factors associated to the half C-terminal region of Gemin5 included proteins known to be involved in translation regulation, RNA stability, and SMN complex, such as poly(A) binding protein cytoplasmic 1 (PABPC1), insulin-like growth factor 2 mRNA-binding protein 1 (IGF2BP1), Fragile X Messenger Ribonucleoprotein 1, or Gemin4. PABPC1 and IGF2BP1 were also detected bound to the N-terminal fragments regardless of the oligomerization capacity of the protein, although with a lower *Z* score. Furthermore, factors associated to G5_845–1508_ such as Gemin4, Gemin3, and SMN were detected with G5_1–1287_ with high *Z* score, but not with G5_1–739_, highlighting a distinctive interactome of the N-terminal WD40 repeats region (WD1–WD2), the extended N-terminal region (WD1–WD2–TPR), and the C-terminal half of the protein (TPR–RBS).

The observation that RNA-binding can contribute to Gemin5 oligomerization (Supplementary Fig. S3) led us to identify the proteins bound to G5_845–1508_ in the presence or absence of RNase treatment in the second step of the TAP procedure. The results revealed that many factors were lost, as only 40% were resistant to the treatment (Supplementary Fig. S4A). Interestingly, GO classification of factors identified with −log(*p* value) >3 which were lost upon RNase treatment (156) included the SMN complex as well as many RBPs involved in splicing, RNA stability, and translation regulation (Supplementary Fig. S4B, C). In contrast, the factors (106) classified into networks resistant to the RNase treatment included a small number of RBPs. The resistant factors are mostly ribosomal proteins, and several factors involved in immune response, stress response, and cell–cell adhesion processes (Supplementary Fig. S4B, D).

Since the C-terminal end of Gemin5 (G5_1287–1508_) contains the non-canonical RBD shown to fold as a compact pentamer [40], we sought to investigate whether the interactome observed with the oligomerizing-proficient protein G5_845–1508_ that folds in vitro as a dimer of pentamers was shared with the oligomerization-defective region (G5_1287–1508_) (Supplementary Fig. S5A). The specific interactome identified with G5_845–1508_, containing both the TPR and the RBS domains, comprised the terms Ribosome, Splicing, SMN complex, Translation regulation, and RNA stability (Supplementary Fig. S5B), as it does the interactome shared by other dimerization proficient regions (Fig. 2B). In contrast, the interactome shared by G5_845–1508_ and G5_1287–1508_ showed lower statistical significance in the common GO terms (Ribosome, Translation regulation, Splicing, and RNA stability) and higher diversity of terms, as Retina homeostasis, Stress response, Protein modification, and Immune response (Supplementary Fig. S5C).

Taken together, these results confirm that the interactome associated to Gemin5 is largely dependent upon its oligomerization properties.

### The association with general RNA-binding proteins requires Gemin5 oligomerization

Next, we study whether the Gemin5 associated proteins performing RNA-dependent processes related to splicing, translation regulation, polyadenylation, and RNA stability would be dependent on Gemin5 oligomerization. A heatmap plot of the RBPs identified with the different TAP-tagged domains of Gemin5 indicated that the TPR-containing regions (G5_1–1287_, G5_845–1508_) displayed overall greater association of factors involved in RNA biology processes compared to those lacking the TPR domain (G5_1–739_) or having a defective dimerization domain (G5_845–1508_AxE) (Supplementary Fig. S5D). Interestingly, the associated RBPs clustered in two groups according to their GO terms. Group I comprised factors related to splicing regulation, spliceosome assembly, and polyadenylation. Group II included regulation of translation and RNA stability. Notably, the most N-terminal end G5_1–739_ denoted a strong decrease in protein interaction, compared to G5_1–1287_. However, the decrease was even stronger in the oligomerization-defective mutant G5_845–1508_AxE compared to G5_845–1508_. Furthermore, most of the RBPs lost in G5_845–1508_AxE and in G5_845–1508_ treated with RNase are coincident (Supplementary Fig. S4B), reflecting the relationship between Gemin5 RNA-dependent functions and its oligomerization capacity. These data reinforce the idea that Gemin5 oligomerization driven by the TPR moiety determines the interaction with most of these RBPs.

### Gemin5 oligomerization is required for interaction with proteins of the SMN complex

**Fig. 3:**
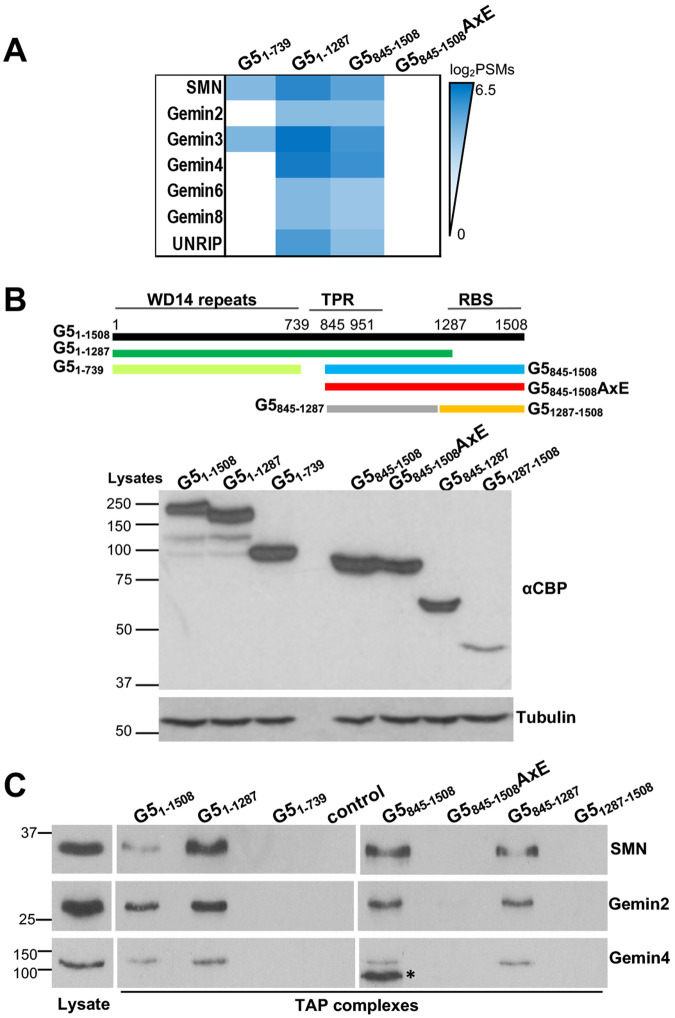
Validation of SMN complex members association with Gemin5 domains. **A** Heatmap representing the interactions of SMN complex members with Gemin5 domains observed by mass spectrometry. **B** Schematic of functional domains of Gemin5 and the proteins used in the assay. HEK293 cells transfected with plasmids expressing the TAP constructs were lysed, and protein expression was followed by immunoblot using anti-CBP antibody. Tubulin was used as a loading control. **C** Coimmunoprecipitation of the Gemin5 domains with representative members of the SMN complex (SMN, Gemin2, and Gemin4). After TAP purification, the presence of SMN, Gemin2, and Gemin4 proteins was analyzed in three independent experiments by WB using specific antibodies.

The distinctive interactome pattern of the oligomerization-proficient regions (G5_1–1287_ and G5_845–1508_) with members of the SMN complex (Fig. 3A) prompted us to confirm biochemically whether Gemin5 oligomerization was required for the interaction with these proteins individually. Expression of the proteins G5_1–1508_, G5_1–1287_, G5_1–739_, G5_845–1508_, G5_845–1508_AxE, G5_845–1287_, and G5_1287–1508_ was verified by WB using anti-calmodulin binding protein (CBP) epitope tag antibody (Fig. 3B). Then, the TAP complexes prepared from HEK293 cells expressing each of these proteins were examined by WB with specific antibodies against three members of the SMN complex. A control lysate expressing only the TAP tag was negative in all cases (Fig. 3C). The proteins SMN, Gemin2, and Gemin4 were detected associated to G5_1–1508_, G5_1–1287_, G5_845–1508_, and G5_845–1287_ TAP complexes. In contrast, the proteins lacking the TPR domain (G5_1–739_ and G5_1287–1508_) and the oligomerization-defective mutant G5_845–1508_AxE failed to detect any of these proteins using the same antibodies, in agreement with the *Z* score value obtained for oligomer associated factors (Supplementary Table S1). Thus, we conclude that oligomerization of Gemin5 is a critical feature required for interaction with members of the SMN complex.

### The predominant cytoplasmic localization of Gemin5 is independent of oligomerization

**Fig. 4:**
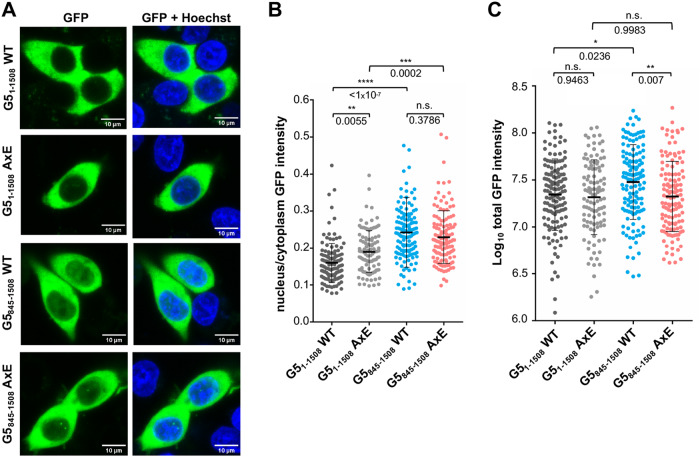
Subcellular localization of Gemin5. **A** Nucleus–cytoplasm distribution of G5_1–1508_GFP WT, G5_1–1508_GFP AxE, G5_845–1508_GFP WT, and G5_845–1508_GFP AxE. Representative images of transfected cells are shown (nucleus stained with Hoechst). **B** Diagram depicts the signal (integrated density value) of nucleus/cytoplasm ratio emitted from cells expressing the fluorescent GFP protein. Differences of the average intensity ratio between G5_1–1508_GFP WT (*n* = 134), G5_1–1508_GFP AxE (*n* = 107), G5_845–1508_GFP WT (*n* = 136), and G5_845–1508_GFP AxE (*n* = 128) were analyzed using one-way ANOVA followed by Tukey’s HSD test. **C** Total fluorescence intensity (log_10_ integrated density value) of cells expressing G5_1–1508_GFP WT, G5_1–1508_AxE GFP, G5_845–1508_GFP WT, and G5_845–1508_GFP AxE. Values represent the mean ± SD. Asterisks denote P values (n.s. not significant, *P < 0.1, **P < 0.01, ***P > 0.001, ****P < 0.0001).

Gemin5 is a primary cytoplasmic protein [27], abundantly found outside from SMN complexes in nuclear extracts [42]. Therefore, we wondered whether the lack of oligomerization capacity of the G5_845–1508_AxE mutant and the changes in the interactome could result from a different subcellular localization. To this end, we assessed by confocal microscopy the nuclear/cytoplasmic localization of the full-length G5_1–1508_ and the G5_845–1508_ WT proteins, as well as the AxE mutant of both constructs using GFP-tagged proteins (Fig. 4A). Measurement of the nucleus/cytoplasm intensity revealed a modest increase for the AxE mutant in the full-length protein. This difference was not observed in the C-terminal half of the protein (Fig. 4B). Nonetheless, quantification of the total GFP intensity revealed a higher intensity of the G5_845–1508_ WT protein compared to the full length and the G5_845–1508_AxE (Fig. 4C), suggesting that the levels of G5_845–1508_ WT are slightly higher than all the others. Collectively, these data show that loss of protein oligomerization does not affect the subcellular localization of Gemin5.

### The contiguous WD40–TPR domains mediate Gemin5–ribosome interaction

**Fig. 5:**
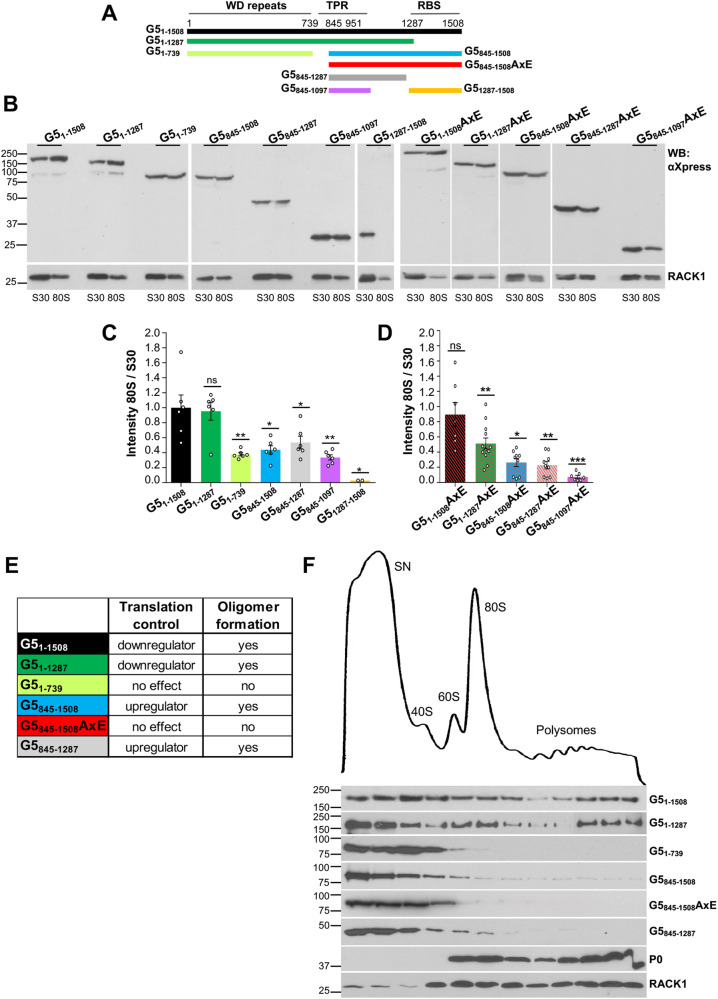
Association of Gemin5 domains with native ribosomes and polysomes. **A** Schematic representing the proteins used in the analysis. **B** HEK293 cells transfected with Xpress-His wild type or the mutant A951E constructs expressing Gemin5 domains were processed to isolate native ribosomes (80S) in independent experiments (*n* = 2 for G5_1287–1508_; *n* = 6 for G5_1–1508_, G5_1–1287_, G5_1–739_, G5_845–1508_, G5_845–1287_, G5_845–1097_; *n* = 7 for G5_1–1508_AxE, G5_845–1097_; *n* = 9 for G5_845–1508_AxE, G5_845–1287_; *n* = 12 for G5_1–1287_AxE). The protein levels present in S30 fractions (cytoplasmic lysates) and 80S ribosomes were determined using anti-Xpress antibody. Anti-RACK1 was used as loading control. **C** Representation of Xpress-G5 protein intensity in native ribosomes (80S) divided by the intensity present in total lysates (S30), relative to those of G5_1–1508_. Values represent the mean ± SEM, asterisks denote *P* values (ns not significant, *P < 0.05, **P < 0.01, ***P < 0.001). **D** Histogram represented as in (**C**). Ratio for the AxE mutants was made relative to its wild-type version value in each case. **E** Summary table indicating the effect on translation and the oligomerization capacity of the Gemin5 domains. **F** Polysome profiles were prepared in 10–50% sucrose gradients loaded with total lysates of HEK293 cells expressing the Xpress-His proteins G5_1–1508_, G5_1–1287_, G5_1–739_, G5_845–1508_, G5_845–1508_AxE, or G5_845–1287_. The fractions of a representative polysome profile corresponding to the supernatant (SN), 40S and 60S ribosomal subunits, 80S monosomes and polysomes are indicated. Xpress-His Gemin5 versions and the ribosomal proteins P0 (60S) and RACK1 (40S), were analyzed all along the gradient fractions from two independent experiments by WB using specific antibodies.

Next, to test whether the differential interaction of Gemin5 with the ribosomal proteins observed by mass spectrometry (Supplementary Fig. S6) was dependent upon the presence of an active oligomerization domain, we prepared S30 cytoplasmic lysates and 80S native ribosomes from cells expressing Xpress-tagged forms of the Gemin5 regions (Fig. 5A). The mutation A951E was also included in the corresponding domains. Expression of the G5-Xpress-tagged proteins was verified by immunoblotting using anti-Xpress antibody (Fig. 5B). Similarly, the presence of ribosomes in S30 and 80S fractions was verified using anti-RACK1. Then, the interaction of Gemin5 with the native ribosomes was measured as the 80S/S30 intensity, setting as 1.0 the ratio observed for the full-length G5_1–1508_ protein (Fig. 5C), previously shown to bind native ribosomes in vitro using purified protein [43]. Full capacity to interact with 80S native ribosome was only observed in the WD40–TPR-extended protein G5_1–1287_ (Fig. 5C). The other versions of the protein (G5_1–739_, G5_845–1508_, G5_845–1287_, and G5_845–1097_) revealed a significant reduction in ribosome binding. The decrease was even stronger for the most C-terminal part of the protein (G5_1287–1508_) which lacks the WD40 repeats and the TPR domain. These results indicate that robust interaction of Gemin5 with the ribosome in the cell requires both, the WD40 repeats domain and the TPR dimerization moiety of Gemin5.

We also analyzed the presence in native ribosomes of the defective AxE oligomerization mutants (Fig. 5B). With the exception of G5_1–1508_AxE, binding with native ribosomes was significantly reduced (Fig. 5D), in accordance with their oligomerization properties (Fig. 1D). Although the lack of effect of the mutant G5_1–1508_AxE remains unknown, we reasoned that it can be related to its ability to oligomerize (Fig. 1D). Therefore, we conclude that the oligomerization capacity determines ribosome binding ability.

### Gemin5–polysomes interaction underscores the need of both WD40 repeats and TPR domains for downregulation of translation

Different regions of Gemin5 exhibit distinct effects on translation regulation and oligomerization ability (Fig. 5E). For instance, the full-length G5_1–1508_ and the extended N-terminal region (G5_1–1287_) negatively regulate global translation [43], whereas versions containing the TPR and RBS domains (G5_845–1508_ and G5_845–1287_) exert a positive effect on protein synthesis [38]. In contrast, the oligomerization-deficient regions, G5_845–1508_AxE [38] and G5_1–739_ (Supplementary Fig. S7A, B), lose the regulatory effect on global translation. Next, to examine the impact of Gemin5 oligomerization on the translation of selective mRNA targets we evaluated the translation efficiency of the luciferase reporters harboring the TOP sequence (L32 WT TOP) at the 5′end (Supplementary Fig. S7C), and histone stem-loop sequence (luc-hSL) at the 3′end (Supplementary Fig. S7D) [23]. The results revealed that the oligomerization-proficient protein (G5_845–1508_) upregulates translation relative to control cells, while the oligomerization-deficient (G5_845–1508_AxE) loses this effect (Supplementary Fig. S7C, D). Interestingly, in the case of the specific mRNAs (TOP and hSL), G5_845–1508_, and G5_1–1508_ behaved the same way. These results reinforce the relevance of Gemin5 oligomerization to perform its role in the regulation of translation.

To test the requirement of the WD40 repeats and the TPR module for the interaction with translation-competent ribosomes, and therefore its functional impact on translation control, we analyzed the presence of Gemin5 protein versions in the fractions of polysome profiles obtained with lysates expressing each of these Gemin5 regions. Detection of RACK1 denoted the presence of 40S subunits and 80S ribosomes in the profile, whereas ribosomal protein P0 revealed the 60S subunits and 80S ribosomes (Fig. 5F). Both, the full-length protein and the extended N-terminal version (G5_1–1287_) were observed all along the fractions of the profile, including heavy polysomes (Fig. 5F), in agreement with previous data [43]. The remaining Gemin5 versions were detected in the supernatant (SN) and the 40S ribosomal subunit, showing a strong decrease on the 60S subunit and the 80S ribosomes (Fig. 5F). Furthermore, in comparison to the full-length protein, a weak signal of G5_845–1508_ was detected in polysomes. The proteins G5_1–739_, G5_845–1508_AxE, and G5_845–1287_ remained undetected in polysomes (Fig. 5F), consistent with the reduced presence of these proteins in 80S ribosomes (Fig. 5A, B). Therefore, these results establish a reciprocal link between the downregulation of global protein synthesis and the presence of Gemin5 regions containing both the WD40 repeats and the TPR domain in heavy polysomes.

## Discussion

Gemin5 is a multifunctional protein impacting on fundamental RNA-dependent processes, including spliceosome assembly and translation regulation. The arrangement of this protein in distinct functional domains promotes the interaction with snRNAs, ribosomes and various mRNAs translated via cap-dependent or cap-independent mechanisms [13, 20, 21]. Interestingly, the central region of the protein harbors a potent dimerization domain, which in combination with a pentamer structure at the most C-terminal end adopts a decamer architecture [38, 40]. Importantly, this structural conformation determines the RNA-binding capacity and translation regulation properties of the C-terminal half of the protein [40, 41]. Here we show that the oligomerization state of Gemin5 is at the basis of its capacity to interact with (1) factors involved in spliceosome assembly, and (2) its association with heavy polysomes, supporting a dedicated role in translation regulation. Failure to oligomerize, either as we show by absence or mutation of the TPR module, leads to lack of association with multiple factors, including many RBPs, members of the SMN complex, and particularly, ribosomal proteins and translation regulation factors (Fig. 2). Gross changes in the Gemin5 interactome are consistent with previous data showing that variants in conserved residues of the TPR moiety, which induce conformational changes of the dimerization module, impact on RNA-dependent pathways [32].

The functional implications of oligomerization domains on RBPs are well documented. The oligomerization state of proteins impinges on their direct and indirect targets, their dynamic range of activities including autoregulation, as well as their ability to sense intracellular and extracellular signals. In particular, the dimerization domain of the SMN protein leverages the assembly of the entire complex depending upon the phosphorylation state of its YG motif [44, 45]. Many other RBPs contain oligomerization motifs that regulate their state, and consequently their function [46–48]. For instance, the oligomerization capacity of the splicing factor RNA binding protein mRNA processing factor regulates the splicing program of smooth muscle cells in response to external signals [49].

Curiously, Gemin5 comprises a singular combination of structural domains, uncommonly found juxtaposed in RBPs though individually are found in proteins participating in a broad range of cellular processes. The domains WD1–WD2-TPR are placed next to each other in the yeast vesicle coating proteins ß’-COP and α-COP of the COPI complex, and Sec31 of the COPII complex [50, 51]. The helical regions of the mentioned proteins are preceded by N-terminal WD40 domains, as it also happens in Gemin5 [35, 36, 38]. This observation highlights a modular arrangement evolved into proteins with unrelated functions. On the other hand, the dimer of pentamers architecture of the C-terminal half of Gemin5 was found in NLR family pyrin domain containing 3 (NLRP3) and cyanase [40], two proteins lacking functional similarity with Gemin5. NLRP3 detects exogenous pathogenic invasions and endogenous cellular damage, and responds by forming the NLRP3 inflammasome, activating caspase-1 [52]. Understanding the functional relevance of this combination of structural domains in Gemin5 has remained elusive, though it confers a way to regulate two key steps of the RNA life, spliceosome assembly and translation regulation, with the same protein.

**Fig. 6:**
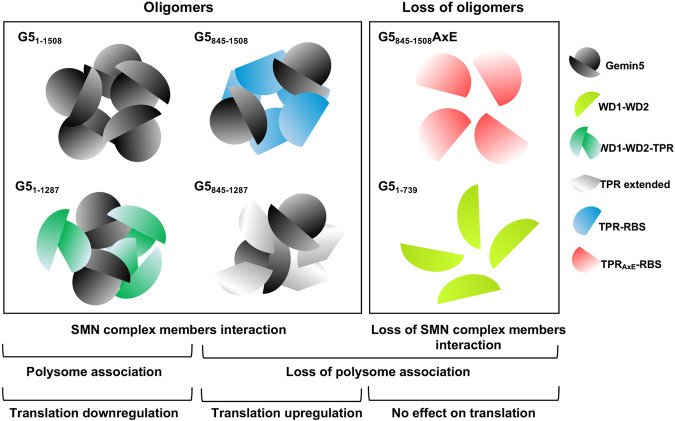
Gemin5 oligomerization capacity elicits distinctive interaction with ribosome machinery and with SMN complex members. Summary of the distinctive properties of the oligomerizing proteins (G5_1–1508_, G5_1–1287_, G5_845–1508_ and G5_845–1287_) regarding the interaction with members of the SMN complex and the association with polysomes. Only G5_1–1508_ and G5_1–1287_, which harbor the WD40 repeats and the TPR domains, associate with polysomes and elicits a downregulatory role in global protein synthesis. In contrast, the G5_845–1508_ protein lacking the WD1–DW2 domains upregulates translation, establishing a marked difference with the full-length protein. The proteins lacking oligomerization properties, G5_1–739_ and G5_1–1508_AxE, fail to interact with members of the SMN complex, and do not elicit negative or positive effects on translation. Our working model hypothesizes that Gemin5 contributes to diverse functions through the assembly of distinctive cellular condensates. The Gemin5 interactors identified with the different domains of the protein include multiple RBPs, as a function of the oligomerization capacity of the corresponding domain, and presumably the conformational architecture of the oligomer. This property could facilitate a specific role of Gemin5 in the assembly of distinct ribonucleoprotein complexes, as described in translation regulation and spliceosome assembly.

Cumulative data support the view of Gemin5 performing distinct roles outside of the SMN complex, contributing to RNA-dependent processes beyond spliceosome assembly [25]. This functional versatility is reflected in the Gemin5 interactome, which depends upon the mutations present on the TPR and the RBS regions [53]. Our current study shows that the Gemin5 interactome is closely linked to its oligomerization capacity as revealed by the loss of most of the associated factors, including members of the SMN complex (Fig. 6), in both, the G5_1–739_ and the defective mutant G5_845–1508_AxE. In agreement with this, the Gemin5 interactors identified in our study include multiple RBPs that participate in RNA-dependent processes such as splicing regulation, polyadenylation, RNA stability, and translation regulation, among others. The possibility that these interactions are stabilized by RNA bridges is likely, as indicated by the reduced Gemin5 recruitment observed following RNase treatment by G5_845–1508_ (Supplementary Fig. S3C), as well as the loss of factors bound to G5_845–1508_ following RNase treatment (Supplementary Fig. S4B). Additional functions of Gemin5 such as its potential contribution to assemble diverse type of cellular condensates can be deduced from its dual capacity to interact with various RNAs and multiple RBPs [21, 23, 40, 43]. This property combined with the oligomerization capacity could facilitate a specific role of Gemin5 in the assembly of distinct ribonucleoprotein complexes, as already reported for translation regulation and spliceosome assembly (Fig. 6).

Previous data suggested a ribosome stalling function of Gemin5 in connection to the downregulatory role of global protein synthesis [43]. Here we have found that the translation downregulatory function is linked to the presence of both the WD40 repeats and the TPR module, since only proteins carrying both of these domains are detected in heavy polysomes (Fig. 5F). Therefore, it is inferred that polysome association requires protein oligomerization but also the WD1–WD2 architecture [35]. This conclusion agrees with the observation that the N-terminal region of the protein (G5_1–739_) is sufficient to bind the large ribosomal subunit in vitro [43]. However, oligomerization-proficient versions of the protein lacking the WD40 repeats behave as upregulators of translation [22, 38], presumably caused by a different macromolecular interactome (Fig. 2), and a distinct three-dimensional architecture. Although the structure of the N-terminal region encompassing the WD40 repeats domains [35–37] and the C-terminal region including the TPR module and the RBS domains [38, 40] have been solved, the 3D structure of the full-length protein and the stoichiometry of the monomer entities in the oligomers remains unknown. This hypothesis also conveys the idea that Gemin5 fragments lacking WD40 repeats domain but maintaining the oligomerization capacity partially highjack the endogenous Gemin5, as supported by the recruitment of the endogenous Gemin5 protein (Fig. 1). Along this view, the differential effect in translation of the oligomerization-proficient fragment G5_845–1508_ could be a consequence of the loss of translation repression induced by the endogenous Gemin5 (Fig. 6). This is in contrast to the interaction with members of the SMN complex, where just the active oligomerization module placed in G5_845–1287_ appears to promote the association with these proteins (Fig. 3).

The involvement of Gemin5 in diverse cellular processes, beyond spliceosome assembly, has been supported by different observations. First, Gemin5 is present in 30S complexes with Gemin3 and Gemin4, and also in 20S outside of the SMN complex, apart from the 40S–80S complexes with SMN and Gemin2 [27]. Second, Gemin5 nuclear bodies do not colocalize with coilin, indicative of its absence in Cajal bodies [42]. Third, the increased association of U1 and U1A with Gemin5 in P bodies in SMN-deficient cells suggested a role in unassembled U1 snRNA disposal [54]. Furthermore, the protein colocalizes with eIF4G in arsenite-induced stress granules [55]. Our current study shows that the oligomerization-defective mutant G5_845–1508_AxE does not interact with members of the SMN complex, and also that the ratio of nuclear/cytoplasm intensity remained similar to the WT (Fig. 4A, B). Thus, the predominant cytoplasmic localization of Gemin5 reinforces the cytoplasmic functions of this essential protein.

The observed reorganization of the Gemin5 interactome as a function of the modular arrangement used to capture associated factors led us to suggest the involvement of distinct macromolecular architectures in proteome association, and therefore for protein function. In support of this possibility, differences in the association with proteins performing dedicated functions in RNA metabolism, such as G3BP Stress Granule Assembly Factor 2, La Ribonucleoprotein 1, or YTH N6-methyladenosine RNA-binding protein F1, are strictly dependent on the oligomerization domain of the protein (Supplementary Fig. S5D). Furthermore, recent works have reported the identification of Gemin5 in protein aggregates related to stress response [55–57], as well as in various complexes involved in viral infection and cancer progression [58–60]. Understanding how these different functions are spatially and temporally coordinated awaits further investigations.

Together, the data presented here support the involvement of Gemin5 oligomerization in two critical aspects of RNA metabolism, namely interaction with components of the SMN complex and association with heavy polysomes. The observed differential requirement suggests the relevance of protein architecture for the interaction with each of these macromolecular complexes.

## Materials and methods

### Constructs

The plasmids pcDNA3-G5FLAG [61], pcDNA3-Xpress-G5_845–1508_, pcDNA3-Xpress-G5_1287–1508_, pETM11-RBS1 [39], pcDNA3-Xpress-Gemin5, pcDNA3-NTAP-G5_1–1287_, pcDNA3-CTAP-G5_1287–1508_ [43], pcDNA3-Xpress-G5_845–1508_-A951E, pcDNA3-Xpress-G5_845–1287_, pcDNA3-Xpress-G5_845–1287_-A951E, pcDNA3-CTAP-G5_845–1508_, and pcDNA3-CTAP-G5_845–1508_-A951E [38] were previously reported. The plasmid pETM11-G5_845–1287_ was generated by inserting the PCR product in the vector pETM11 via BamHI–NotI using standard procedures. The construct pcDNA3-NTAP-G5_1–1508_ was obtained in two steps. First, the EcoRI site was regenerated in plasmid pcDNA3-NTAP-G5_1–1287_ by QuikChange mutagenesis (Agilent Technologies). Next, the DNA fragment encoding the C-terminus of Gemin5 was inserted via EcoRI–NotI. The plasmid pcDNA3-CTAP-G5_845–1287_ was generated by inserting the PCR product via NotI–PacI in pcDNA3-CTAP [62]. pGemin5-GFP was a kind gift of S. S. Bradrick. The construct peGFP-G5_845–1508_ was obtained by inserting the PCR product via XhoI–BamHI in the plasmid peGFP-N1-ARF5 [63]. The constructs pcDNA3-Xpress-G5_1–1287_, pcDNA3-Xpress-G5_1–739_, pcDNA3-NTAP-G5_1–739_, pcDNA3-Xpress-G5_845–1097_, pcDNA3-Xpress-G5_1–1508_-A951E, pcDNA3-Xpress-G5_1–1287_-A951E, pcDNA3-Xpress-G5_845–1097_-A951E, peGFP-G5_1–1508_-A951E, and peGFP-G5_845–1508_-A951E were obtained by QuickChange mutagenesis. The primers used in each case are indicated in Supplementary Table S2. All constructs were confirmed by DNA sequencing (Macrogen).

### Cell culture and pull-down assays

HEK293 cells were cultured in Dulbecco’s Modified Eagle Medium (DMEM) supplemented with 5% fetal calf serum. Monolayers grown at 80% confluency were co-transfected with the plasmid that expresses G5_1–1508_-FLAG and each of the Xpress-His constructs (Xpress-His-control, G5_1–1508_, G5_1–1508_AxE, G5_1–1287_, G5_1–1287_AxE, G5_1–739_, G5_845–1508_, G5_845–1508_AxE, G5_845–1287_, G5_845–1287_AxE, or G5_1287–1508_) using Lipofectamine LTX (Thermo Fischer Scientific). Cells were harvested 24 h post transfection (hpt), except cells expressing G5_1287–1508_ which were harvested 48 hpt.

For PD assays cell lysates were prepared in lysis buffer (10 mM Tris-HCl pH 7.5, 20 mM NaCl, 10 mM KCl, MgCl_2_ 5 mM, NP40 0.1%, and protease inhibitors (Roche)). The concentration of total protein in the lysates (Input) was determined by the Bradford assay (Bio-Rad). The Ni-NTA agarose resin (25 µl) was equilibrated with lysis buffer (without NP40) and incubated with the corresponding cell lysate (the volume containing 1 mg of total protein) at 4 °C, 1 h in a rotating wheel. The tubes were centrifuged to remove the supernatant (14,000 rpm 4 °C 3 min) and the pellets were washed three times with washing buffer (10 mM Tris-HCl pH 7.5, 20 mM NaCl, 10 mM KCl, 5 mM MgCl_2_, 10 mM imidazole, and protease inhibitors). The pellets were resuspended in 30 µl of elution buffer (10 mM Tris-HCl pH 7.5, 20 mM NaCl, 500 mM imidazole). For protein expression analysis, 20 µl of SDS-loading buffer was added prior to incubate the samples 3 min at 92 °C.

For oligomer detection HEK293 cell lysates expressing the Xpress-His constructs (G5_1–1508_, G5_1–1508_AxE, G5_1–1287_, G5_1–1287_AxE, G5_1–739_, G5_845–1508_, G5_845–1508_AxE, G5_845–1287_, or G5_845–1287_AxE) were mixed with SDS-loading buffer supplemented, or not, with 5 mM DTT [47], and heated 3 min at 92 °C. Western blot analysis was carried out using anti-Xpress antibody.

### Protein complexes isolation by TAP

HEK293 monolayers grown at 80% of confluency (about 2 × 10^7^ cells) were transfected with the TAP constructs (TAP control, G5_1–1508_, G5_1–1287_, G5_1–739_, G5_845–1508_, G5_845–1508_AxE, G5_845–1287_, or G5_1287–1508_)_._ Cells were harvested 24 hpt, except G5_1287–1508_ expressing cells which were harvested 48 hpt. The complexes associated with the TAP-tagged proteins were purified as described [43]. An optional treatment to eliminate the factors bound by RNA bridges was performed with RNase A (75 μg/1.5 ml) 30 min at room temperature immediately following TEV protease digestion of the sample G5_845–1508_-TAP. The supernatant of the first purification, treated or untreated with RNase A, was subsequently subjected to a second calmodulin (Agilent Technologies) purification step. Purified proteins were precipitated with 10% trichloroacetic acid, washed with acetone, and dissolved in an SDS-loading buffer. Aliquots (20%) of the protein complexes associated to G5_1–739_-TAP samples were analyzed on silver-stained SDS–polyacrylamide gel electrophoresis (PAGE) (Supplementary Fig. S1) prior to proteomic analysis.

### In-gel digestion and mass spectrometry

The protein bands concentrated in the stacking/resolving gel interface were visualized by Coomassie staining. The gel pieces were distained in acetonitrile:water (CH_3_CN:H_2_O, 1:1), then reduced and alkylated, and digested in situ with sequencing grade trypsin (Promega) [64]. The gel pieces were dried and re-swollen in 50 mM ammonium bicarbonate, pH 8.8, with 60 ng/μl trypsin at a 5:1 protein:trypsin (w/w) ratio. The tubes were kept on ice for 2 h and incubated at 37 °C for 12 h. Digestion was stopped by the addition of 1% TFA. The desalted protein digest was dried, resuspended in 10 μl of 0.1% formic acid, and analyzed by RP-LC–MS/MS in an Easy-nLC II system coupled to an ion trap LTQ-Orbitrap-Velos-Pro hybrid mass spectrometer (Thermo Fisher Scientific), as previously described [32]. Peptides with a *q*-value lower than 0.1 and an FDR < 1% were considered positive identifications with a high confidence level. The number of different proteins identified with >2 unique peptides and high coverage on the overlap of two independent biological replicas for G5_1–739_, G5_1–1287_, G5_845–1508_, and G5_845–1508_AxE, and three replicas for G5_1287–1508_ (Dataset), were 169, 352, 261, 192, and 270_,_ respectively.

### Expression and purification of proteins

Escherichia coli BL21 transformed with plasmids pETM11-RBS1 and pETM11-G5_845–1287_ growing at 37 °C were induced with Isopropyl-β-D-1-thiogalactopyranoside (IPTG) 0.5 mM during 2 h. Bacterial cell lysates were prepared in binding buffer (20 mM NaH_2_PO4, 500 mM NaCl, 20 mM Imidazole) and processed by sonication. Cell debris was eliminated by centrifugation at 16,000 × *g* 30 min at 4 °C twice. The clear lysates were loaded in His-GraviTrap columns (HealthCare) and the recombinant proteins were eluted using Imidazole 500 mM. Proteins were dialyzed against phosphate buffer pH 6.8, 1 mM DTT, and stored at −20 °C in 50% glycerol. The purified proteins were visualized on Coomassie-stained SDS–polyacrylamide gels. Proteins were quantified by UV using extinction coefficients at 205 nm [65].

### RNA electrophoretic mobility shift assay

RNA probes termed SL1 and d5 [66] were uniformly labeled using α^32^P-CTP (500 Ci/mmol), T7 RNA polymerase (10 U), and linearized plasmids (0.5 μg). RNAs were extracted with phenol–chloroform, ethanol precipitated and resuspended in TE to a concentration of 0.04 pmol/μl. RNA integrity and mobility as a single band were examined by 6% acrylamide 7 M urea denaturing gel electrophoresis. RNA-binding reactions were carried out as described [66]. The percentage of the retarded complex was calculated relative to the free probe, run in parallel. GraphPad Prism Software (version 6.01) was used to plot the binding curves by nonlinear regression using the one-site specific binding equation.

### Subcellular fractionation (S30 and native ribosomes)

HEK293 cells, grown to 70–80% confluence, were transfected with Xpress-His constructs (G5_1–1508_, G5_1–1508_AxE, G5_1–1287_, G5_1–1287_AxE, G5_1__–__739_, G5_845__–__1508_, G5_845__–__1508_AxE, G5_845__–__1287_, G5_845__–__1287_AxE, G5_845__–__1097_, G5_845__–__1097_AxE, or G5_1287__–__1508_). Cells were harvested 24 h later (or 48 h in the case of G5_1287__–__1508_), washed with ice-cold PBS and lysed in buffer 1 (15 mM Tris-HCl pH 7.4, 80 mM KCl, 5 mM MgCl_2_, 1% Triton-X-100, and protease inhibitors). Cell debris was discarded by centrifugation at 14,000 × *g* 10 min 4 °C. The supernatant (S30 fraction) was ultracentrifuged at 95,000 rpm during 90 min using the TLA100.2 rotor, yielding the S100 fraction (supernatant) and the native ribosomes (ribosomes plus associated factors). The pellets corresponding to native ribosomes were resuspended in 100 μl of buffer 1. The total protein content in S30 fractions was measured by the Bradford assay and the ribosome concentration was determined as 14 units A260 = 1 mg/ml.

### Polysome fractionation

Polysome profiles were prepared from 2 × 10^7^ HEK293 cells transfected with the Xpress-His constructs G5_1–1508_, G5_1–1287_, G5_1–739_, G5_845–1508_, G5_845–1508_AxE and G5_845–1287_, as described [43]. Briefly, cells were washed with ice-cold PBS containing 100 μg/ml cycloheximide to block ribosomes in the elongation step. Then, cells were lysed with buffer A (15 mM Tris-HCl pH 7.4, 80 mM KCl, 5 mM MgCl_2_, and 100 μg/ml cycloheximide), supplemented with 1% (vol/vol) Triton-X-100, 40 U/ml RNaseOUT (Thermo Fisher Scientific), and protease inhibitors. Cytoplasmic lysates obtained by centrifugation at 14,000 × *g* 10 min at 4 °C, were loaded into a linear 10–50% (wt/vol) sucrose gradient in buffer A and centrifuged at 39,000 rpm with a SW40 Ti rotor 2 h 15 min at 4 °C. Gradients were fractionated by upward displacement with 87% (vol/vol) glycerol using a density-gradient fractionator, monitoring A260 continuously (ISCO UA-5 UV monitor). Fractions (12 fractions of 1 ml) were collected from gradients.

### Translation assays

HEK293 cells were transfected with the plasmid pCAP-luc [67] expressing luciferase in a cap-dependent manner and Xpress-His constructs G5_1__–__1508_, or G5_1–739_. For selective translation assays, cells were transfected with the plasmid harboring the TOP sequence (L32 WT TOP) at the 5′end, or the luc-hSL at the 3′end [23], and those expressing G5_845–1508_ or G5_845–1508_AxE [38]. Cell lysates were prepared 24 hpt in 100 μl lysis buffer (50 mM Tris-HCl pH 7.8, 100 mM NaCl, 0.5% NP40). The protein concentration in the lysate was determined by Bradford assay. Luciferase activity (RLU)/μg of total protein was internally normalized to the value obtained with the empty vector performed side by side. To analyze global protein synthesis, about 10^6^ HEK293 cells transfected with Xpress-His-control, G5_1__–__1508_ and G5_1__–__739_, were radiolabeled with [^35^S]-methionine at the end of the transfection during a 3 h pulse with 10 μCi/well, as described [43]. Cells were kept for 1 h in methionine-free medium before labeling. Proteins were separated on SDS–polyacrylamide gels. Dried gels were used to analyze the incorporation of [^35^S]-methionine in newly synthesized proteins relative to control cells.

### Immunodetection

Equal amounts of total protein were resolved on SDS–PAGE and transferred to a 0.2 μm pore PVDF membrane (Bio-Rad) using a semi-dry electrotransfer (Bio-Rad). Xpress-His-G5 proteins were immunodetected using anti-Xpress (Thermo Fisher Scientific), Gemin5-FLAG, and G5-TAP proteins were detected using anti-FLAG (Merck), or anti-CBP (Abcam) antibodies. SMN, Gemin2, and Gemin4 proteins were detected with anti-SMN, anti-Gemin2, and anti-Gemin4 antibodies (Bethyl), respectively. Immunodetection of tubulin (Merck) was used as loading controls. Antibodies against P0 [68] and RACK1 (Santa Cruz) were used as ribosome markers. The appropriate secondary HRP-conjugated antibodies (Thermo Fisher Scientific) were used according to the instructions of the manufacturer. The uncropped images of the western blots shown in Figs. 1, 3, 5, and Supplementary Figs. S1, S2 are included as Supplementary Material.

### Cell imaging

Approximately 4 × 10^6^ HeLa cells grown at 37 °C, 5% CO_2_ in DMEM supplemented with 10% FBS were electroporated (single pulse, 200 V, 950 µFA, 480 Ω) in 200 µL of media (DMEM Phenol Red-free, 10 mM HEPES pH 8, 37.5 mM NaCl) with 5 µg of DNA expressing G5_845__–__1508_GFP WT (or A951E) or G5_1__–__1508_GFP WT (or A951E), and 20 µg salmon sperm DNA as carrier. Then, 1–2 × 10^6^ transfected cells were seeded in 35 mm glass bottom dishes suitable for confocal microscopy containing phenol red-free culture media. At 24 hpt, nuclei were stained with Hoechst 33342 (20 µM) during 15 min in the incubator. Dishes with the HeLa cells were placed inside a Stage Top Chamber (Okolab) at 37 °C, 5% CO_2_ and visualized with ZEISS LSM800 confocal microscope in vivo. Images were taken using the objective 63× in immersion oil. The imaging settings were identical for all experiments, using ZEN Blue (v3.3). *Z*-stack of each FOV was taken (1.7 µm slice depth, 0.86 µm step, 200 µm of pinhole aperture) encompassing the whole volume of cells. Dimensions of each slice were 1024 × 1024 16-bit pixels (0.62 µm pixel size and 1.03 µs pixel time). GFP was excited using 488 nm wavelength and detected at 500–700 nm emission.

### Statistical analyses

Proteome data were analyzed calculating the *Z* score associated to each interactor for each bait (G5_1–1287_, G5_1–739_, G5_845–1508_, and G5_845–1508_AxE) (Supplementary Table S1) using the average peptide-spectrum match (AvePSM) of the replicates (Dataset). The calculations excluded Gemin5 peptides to prevent the inclusion of bait-related peptides. Interactors scoring >1.96 are significantly enriched in relation to the other protein species (*P* value < 0.05). Complementary to this analysis, we used the CompPASS algorithm [69, 70] to detect high-fidelity interactors characteristic of each region. This analysis compared the data obtained in all TAP-purifications, resulting in a WD-score associated with each bait–prey interaction. Score >1 indicates high-fidelity interactors (95% confidence) (Dataset). All statistical analyses were performed using R version 4.2.3.

Statistical analyses for experimental data, including luciferase activity, protein synthesis, and quantification of the band intensity in western blot analysis and EMSA assays, were performed using data obtained from experiments repeated independently. Values represent the estimated mean ± standard error of the mean. We computed *P* values for a difference in distribution between samples with the unpaired two-tailed Student’s *t*-test. Differences were considered significant when *P* < 0.05. The resulting *P* values were graphically illustrated in figures with asterisks as described in figure legends.

### Image analysis

GFP intensity was quantified from every slice of the *Z*-stacks using ImageJ (v 1.53t). The total intensity of the cell was calculated by adding the integrated density values obtained in each *Z*-stack, distinguishing nucleus and cytoplasm areas using the Hoechst signal. A one-way ANOVA was conducted to assess the mean intensity nucleus/cytoplasm between cells expressing G5_1–1508_GFP WT, G5_1–1508_GFP AxE, G5_845–1508_GFP WT or G5_845–1508_GFP AxE. Post-hoc pairwise comparisons were performed using Tukey’s honestly-significant-difference (HSD) test.

### Bioinformatic analysis

GO analyses were performed by the DAVID database (https://david.ncifcrf.gov) using the overlap of the identified proteins. The significantly enriched biological processes were identified using as a cutoff criteria *P* value < 10^−3^ and a gene count ≥ 3. Clustering analyses were elaborated based on the GO terms associated to each protein. Similarity was calculated using the Euclidean distance between the interactors and clustered using the Ward method.

### Supplementary information

Supplementary Material

Original data files

Supplementary material

Data Set 1

## Data Availability

The mass spectrometry proteomics data generated in this study have been deposited to the ProteomeXchange Consortium via the PRIDE [71] partner repository with the dataset identifier PXD043755 and PXD028959. All data generated or analyzed during this study are included in the manuscript and supporting files.
