# Supplementary material for: Oligomerization regulates the interaction of Gemin5 with members of the SMN complex and the translation machinery
Source: Cell Death Discov. 2024 Jun 28;10:306. doi: 10.1038/s41420-024-02057-5 (PMC11213948; doi:10.1038/s41420-024-02057-5)
Supplement: Supplementary file 1 — Supplementary Material [file 41420_2024_2057_MOESM1_ESM.pdf]

Oligomerization regulates the interaction of Gemin5 with members of the SMN complex and the translation machinery

Rosario Francisco-Velilla, Salvador Abellan, Azman Embarc-Buh, Encarnacion Martinez-Salas*

Supplementary Data

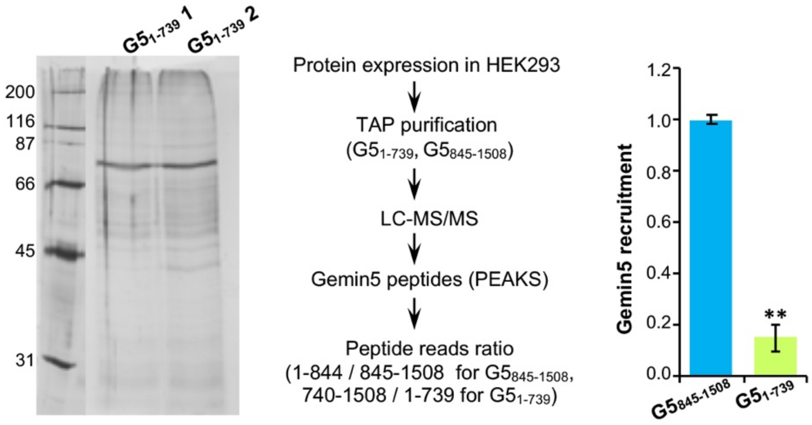

Figure S1. Differential Gemin5 recruitment by the N-terminal and the C-terminal regions of Gemin5. Representative example of a silver stained gel loaded with two biological replicates of the $G5_{1-739}$ protein TAP complexes (left panel). HEK293 cells were transfected with plasmids expressing $G5_{845-1508}$ or $G5_{1-739}$ TAP proteins. Twenty-four hours later, cells were lysed and processed for TAP purification. The endogenous Gemin5 protein associated with $G5_{845-1508}$ or $G5_{1-739}$ was identified by mass spectrometry (central panel). Peptide reads were measured as the number of reads corresponding to the region exclusively present in the endogenous Gemin5 protein (1-844/845-1508 for $G5_{845-1508}$ and 740-1508/1-739 for $G5_{1-739}$) (right panel). Relative values were represented as a histogram and asterisks denote P values (**P <0.01).

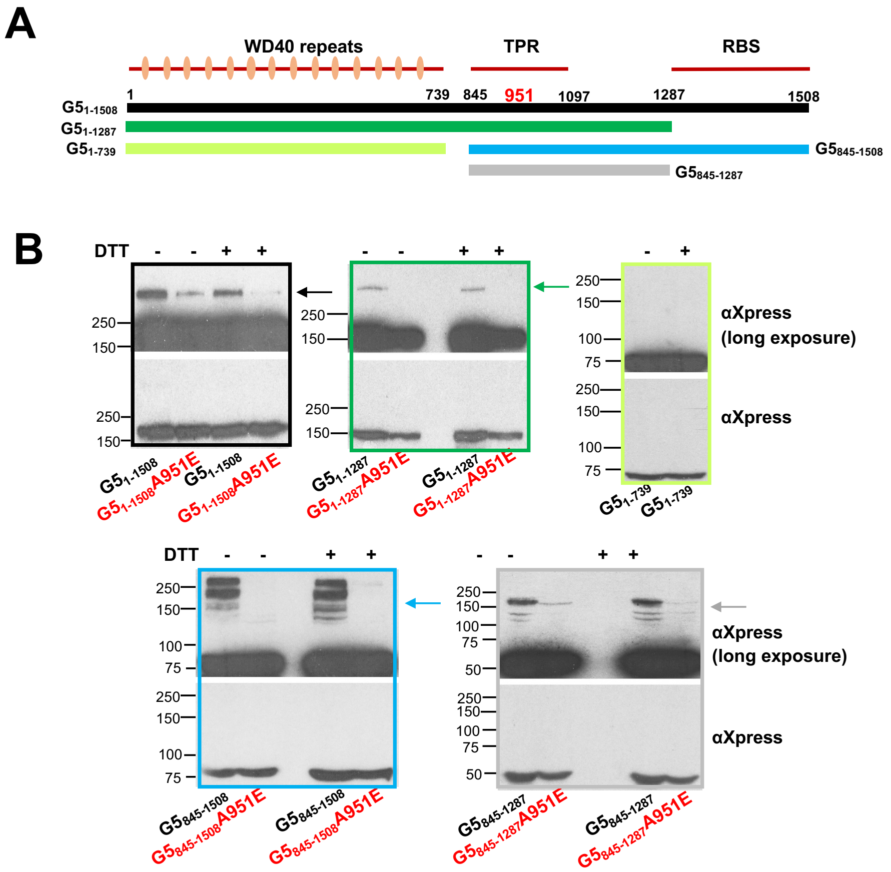

Figure S2. The TPR domain of Gemin5 determines protein oligomerization in the cell. (A) Schematic of Gemin5 domains. The WD40 repeats, the TPR dimerization module, and the non-canonical RNA binding site (RBS) are plotted. The position of amino acids flanking each domain and the dimerization mutation (A951E) are indicated at the top. The different Gemin5 proteins used in the assay are denoted by color bars and the amino acids encompassing each protein are indicated. **(B)** HEK293 cells expressing the corresponding Xpress-His construct ($G5_{1-1508}$, $G5_{1-1508}$AxE, $G5_{1-1287}$, $G5_{1-1287}$AxE, $G5_{1-739}$, $G5_{845-1508}$, $G5_{845-1508}$AxE, $G5_{845-1287}$, or $G5_{845-1287}$AxE) were lysed. For SDS-PAGE, the samples were treated with SDS loading buffer supplemented, or not, with DTT 5 mM, and heated 3 minutes at 92 ºC. Western blot analysis was carried out using anti-Xpress antibody. A light exposure is shown to visualize the Xpress-His construct in each case, and a long exposure is shown to detect high molecular weight bands.

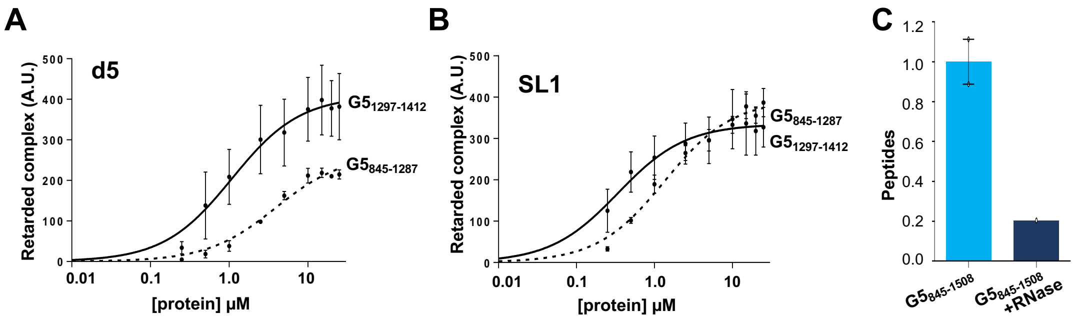

Figure S3. RNA binding capacity of the extended TPR module. (**A-B**) Gel-shift analysis of Gemin5 cognate RNAs with $G5_{845-1287}$ protein in comparison with $G5_{1297-1412}$. The graphs represent the adjusted curves obtained from the quantification (mean ± SD) of three independent gel-shift assays using two RNA probes, domain 5 of the FMDV IRES (d5) **(A)** and the stem-loop 1 (SL1) of Gemin5 mRNA **(B)** incubated with increasing amounts of His-$G5_{845-1287}$ and His-RBS1 proteins. **Gemin5 recruitment by $G5_{845-1508}$ without RNA bridges**. **(C)** The endogenous Gemin5 associated with $G5_{845-1508}$ was identified by mass spectrometry in cells treated or untreated with RNase A after the first TAP purification step. The total reads of the unique peptides corresponding to the residues 1-844 of Gemin5 was made relative to those found in the bait $G5_{845-1508}$.

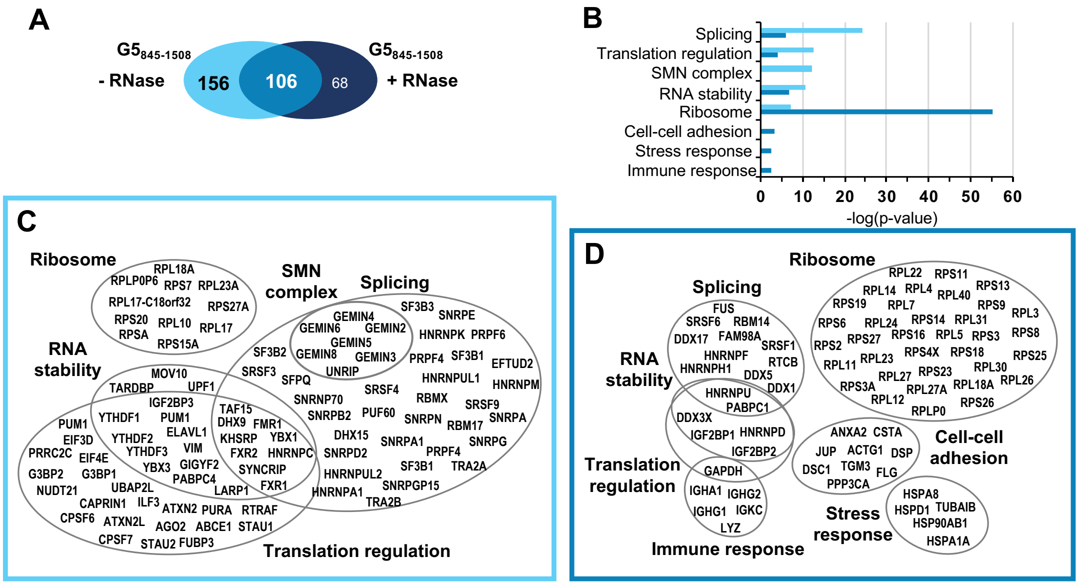

Figure S4. Protein networks interacting with the half C-terminal region of Gemin5 resistant to RNase treatment. (A) Proteins associated to Gemin5 identified by LC-MS/MS with > two unique peptides in two independent biological replicate assays. Venn diagram depicts the number of factors identified bound to $G5_{845\text{-}1508}$ (106) resistant to RNase treatment. **(B)** Bar chart representing the Gene Ontology classification of the identified factors, which are either lost (pale blue) or resistant (blue) to RNase treatment (-log (p-value) >3). **(C,D)** Members of the networks differentially associated to $G5_{845\text{-}1508}$ in the presence or absence of RNase treatment during the purification process.

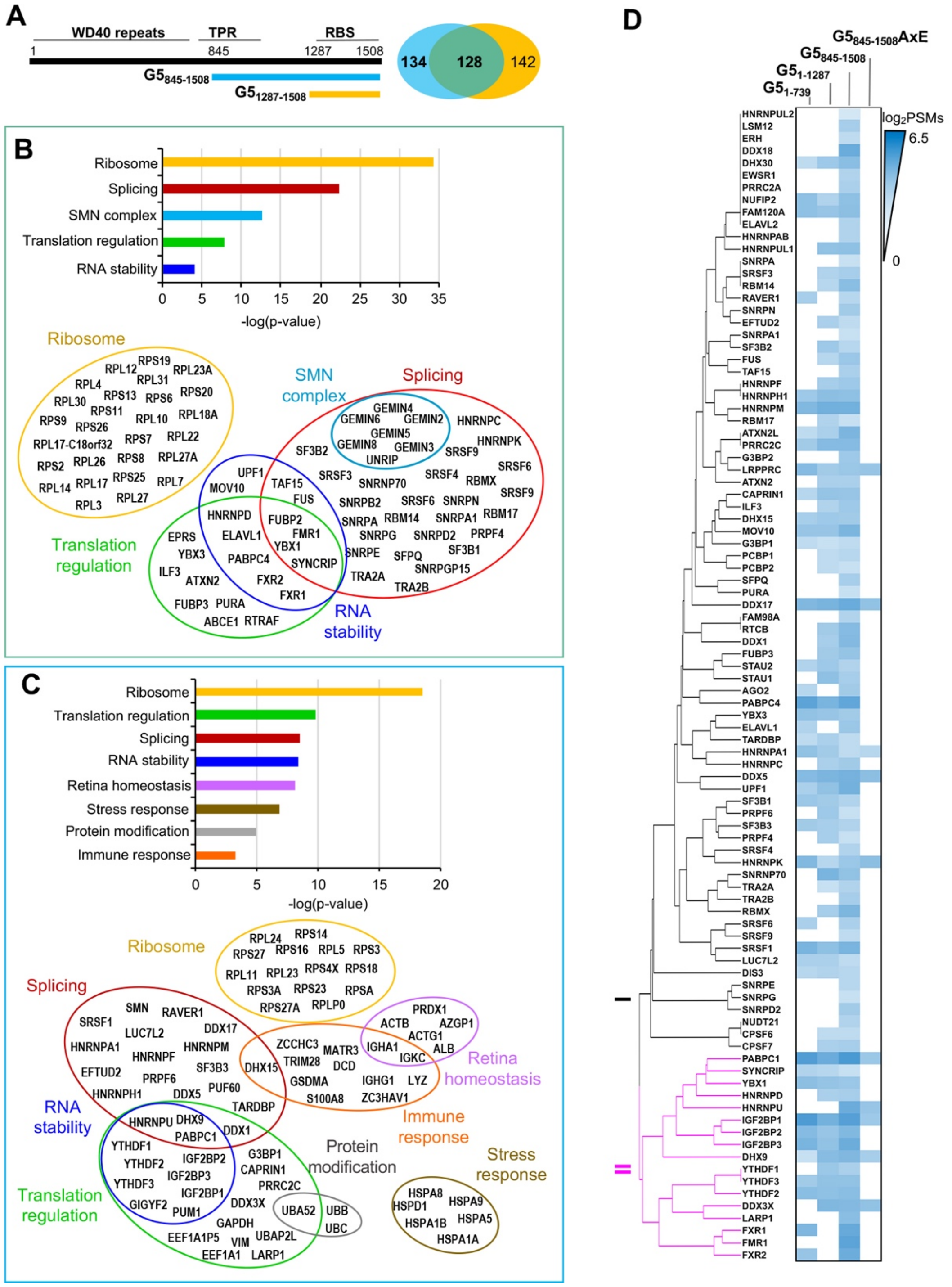

Figure S5. Protein networks interacting with the C-terminal domain of Gemin5. (A) Schematic representing the Gemin5 regions studied in this assay. Venn diagrams indicate the number of factors shared between $G5_{845-1508}$ and $G5_{1287-1508}$ domains. (**B**-**C**) Bar chart representing the Gene Ontology (GO) classification and image showing the name of the factors included in the GO groups for exclusive factors recruited by $G5_{845-1508}$ (134) **(B)**, and the shared factors between $G5_{845-1508}$ and $G5_{1287-1508}$ (128) **(C)**. **(D)** RNA binding proteins interacting with Gemin5 domains. The heatmap depicts the interactors of Gemin5 domains related to RNA cellular pathways observed in the proteomic analysis. The dendogram depicts the clustering of the factors according to their associated GO terms. Group I include proteins

associated to splicing, splicing regulation, spliceosome assembly and polyadenylation. Group II comprises regulation of translation and RNA stability.

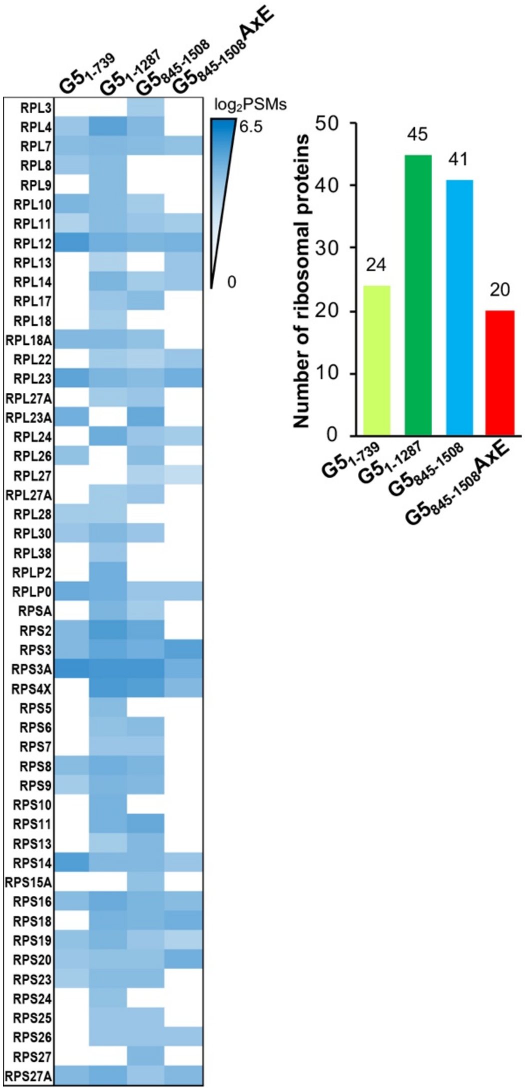

Figure S6. Ribosomal proteins associated with Gemin5 domains. Heatmap depicting the interactions of ribosomal proteins with Gemin5 domains observed in the proteomic analysis. The histogram represents the total number of ribosomal proteins associated to each Gemin5 domain.

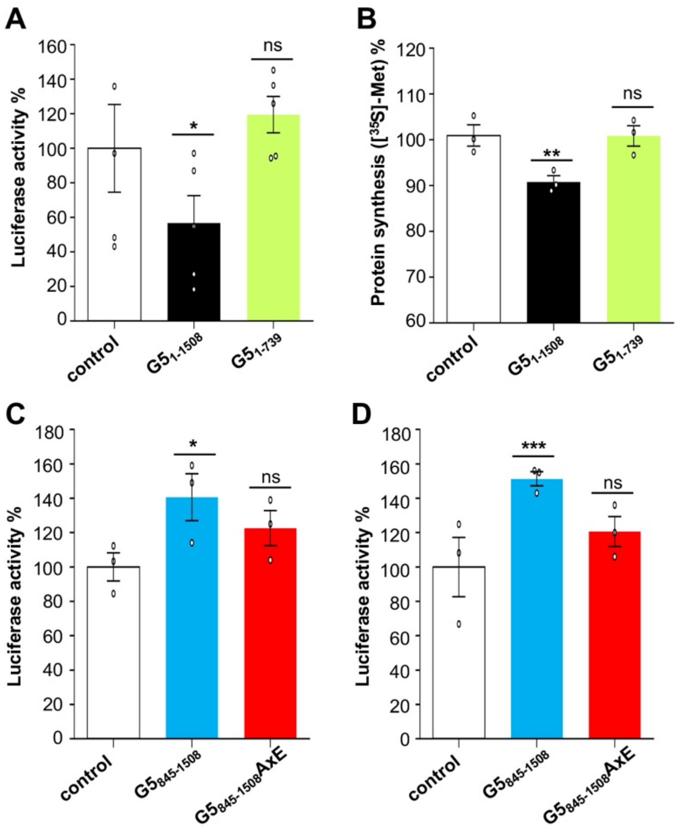

Figure S7. Effect of Gemin5 WD repeats domain on translation. (A) HEK293 cells were transfected with pCAP-luc plasmid and the corresponding Xpress-His construct (control empty vector, $G5_{1-1508}$ and $G5_{1-739}$). Luciferase activity was measured in the cell lysates and the values were normalized to cells expressing the empty vector conducted side by side. **(B)** Histogram depicting the intensity values of [^{35}S]-Met labeled proteins during a 3 h pulse in HEK293 cells expressing the Xpress-His constructs (control empty vector, $G5_{1-1508}$ and $G5_{1-739}$). **Effect of Gemin5 oligomerization on selective translation.** Bar plot depicting the effect of $G5_{845-1508}$ and $G5_{845-1508}$AxE of ribosomal L32 WT TOP **(C)** and histone stem-loop (Luc-hSL) mRNAs translation **(D).** In all cases values represent the mean ± SEM obtained in three independent assays and asterisks denote P-values (ns, not significant, *P < 0.05, **P < 0.01, ***P < 0.001).

Table S1. Z-scores obtained for oligomer-associated factors.

**Interacting protein**	**$G5_{1-739}$**	**$G5_{1-1287}$**	**$G5_{845-1508}$**	**$G5_{845-1508}$AxE**
	No oligomer	Oligomer	Oligomer	No oligomer
PABPC1	**4.20**	**3.29**	**8.32**	0.04
IGF2BP1	**4.63**	**2.39**	**5.94**	0.01
FMR1	-0.01	-0.44	**4.79**	-0.62
PABPC4	**2.91**	**1.96**	**4.17**	-0.24
FXR1	0.86	**0.94**	**3.55**	-0.62
DDX17	0.79	**2.07**	**3.19**	0.26
HNRNPU	0.26	**3.40**	**3.08**	0.10
IGF2BP3	1.05	0.68	**3.03**	-0.62
FXR2	0.09	0.62	**2.98**	-0.62
GEMIN4	-0.27	**5.75**	**2.35**	-0.30
HNRNPM	0.72	**2.60**	**2.35**	0.89
UPF1	-0.24	0.73	**2.25**	-0.62
DDX5	0.56	1.91	**2.20**	0.39
GEMIN3	-0.11	**7.78**	**1.99**	-0.11
EEF1A1	**2.02**	**1.96**	1.26	1.29
SMN	-0.14	**3.56**	0.95	-0.49
SNRP70	-0.67	**1.96**	0.59	-0.62
GIGYF2	-0.40	**2.44**	0.54	-0.62
RPL5	1.02	**3.08**	0.12	0.64

Table S2. DNA constructs and primers.

CONSTRUCT	PRIMER	SEQUENCE
pETM11-G5$_{845\text{-}1287}$	BamHI_G5_845-1287 s	atggatccgctcgttccttgctt
	NotI_G5_845-1287 as	atgcggccgctcaatacagacgcccataa
pcDNA3-NTAP-G5$_{1\text{-}1508}$	G5_EcoRI s	tcgagcggccgcgaattcatacagacgcccata aga
	G5_EcoRI as	tctttatgggcgtctgtatgaattcgcggccgctcga
	EcoRI_G5_1287-1508 s	tgaattctggtggtctctctc
	NotI_G5_1287-1508 as	aagcggccgctcacatacagaaggtctgg
pcDNA3-CTAP-G5$_{845\text{-}1287}$	NotI_G5_845-1287 s	aagcggccgcaccatggctcgttccttgcttcc
	PacI_G5_845-1287 as	cattaattaattcatacagacgcccat
peGFP-G5$_{845\text{-}1508}$	XhoI_G5_845-1508 s	tactcgagatggctcgttccttgcttc
	BamHI_G5_845-1508 as	atggatccgccatacagaaggtctggc
pcDNA3-Xpress-G5$_{1\text{-}1287}$	G5_1-1287stop s	ggagagagaccaccattattcatacagacgcc cataaagaaaaaac
	G5_1-1287stop as	ctttttctttatgggcgtctgtatgaataatggtggtct ctctcc
pcDNA3-Xpress-G5$_{1\text{-}739}$<br>pcDNA3-NTAP-G5$_{1\text{-}739}$	G5_1-739stop s	ctttttgggctatgccttaggttgagagagccg
	G5_1-739stop as	cggctctctcaacctaaggcatagcccaaaaag
pcDNA3-Xpress-G5$_{845\text{-}1097}$	G5_845-1097stop s	gctcccacccagttgtttcacagaagcagctcttggg
	G5_845-1287stop as	cccaagagctgcttctgtgaaacaactgggtggg agc
pcDNA3-Xpress-G5$_{1\text{-}1508}$-A951E<br>pcDNA3-Xpress-G5$_{1\text{-}1287}$-A951E<br>pcDNA3-Xpress-G5$_{845\text{-}1097}$-A951E	G5_TPR_A951E s	ccagctgctggtgccatttccacaaggttgtctgtc
	G5_TPR_A951E as	gacagacaaccttgtggaaatggcaccagcag ctgg
