# Supplementary material for: Oligomerization regulates the interaction of Gemin5 with members of the SMN complex and the translation machinery
Source: Cell Death Discov. 2024 Jun 28;10:306. doi: 10.1038/s41420-024-02057-5 (PMC11213948; doi:10.1038/s41420-024-02057-5)

Fig. 1B

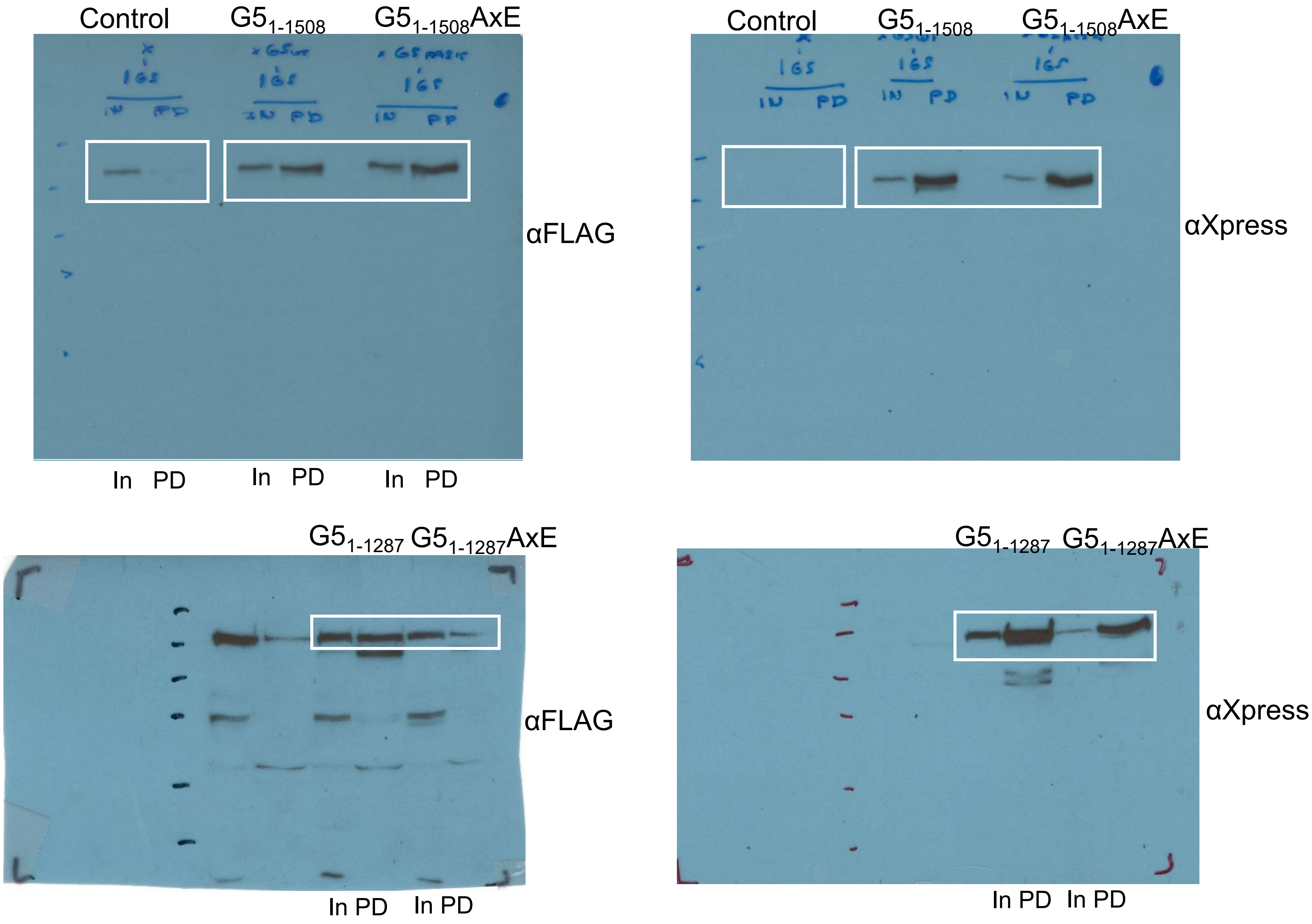
Control
G5 1-1508
G5 1-1508 AxE
αFLAG
In PD In PD In PD
Control
G5 1-1508
G5 1-1508 AxE
αXpress
G5 1-1287 G5 1-1287 AxE
αFLAG
In PD In PD
G5 1-1287 G5 1-1287 AxE
αXpress
In PD In PD

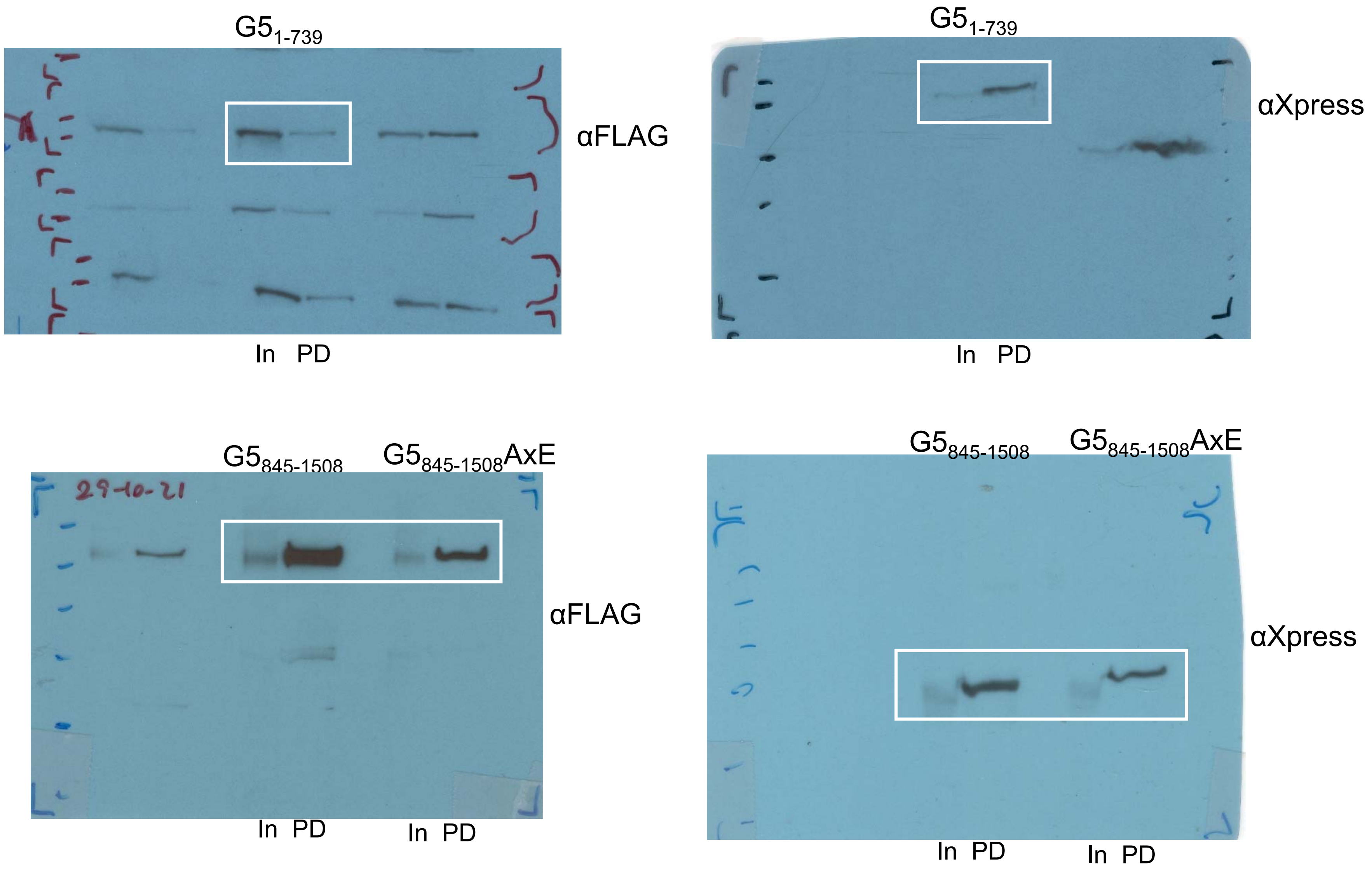

G5$_{1-739}$
αFLAG
In PD
G5$_{1-739}$
αXpress
In PD
29-10-21
G5$_{845-1508}$
G5$_{845-1508}$AxE
αFLAG
In PD
In PD
G5$_{845-1508}$
G5$_{845-1508}$AxE
αXpress
In PD
In PD

Fig. 1B

Fig. 1B

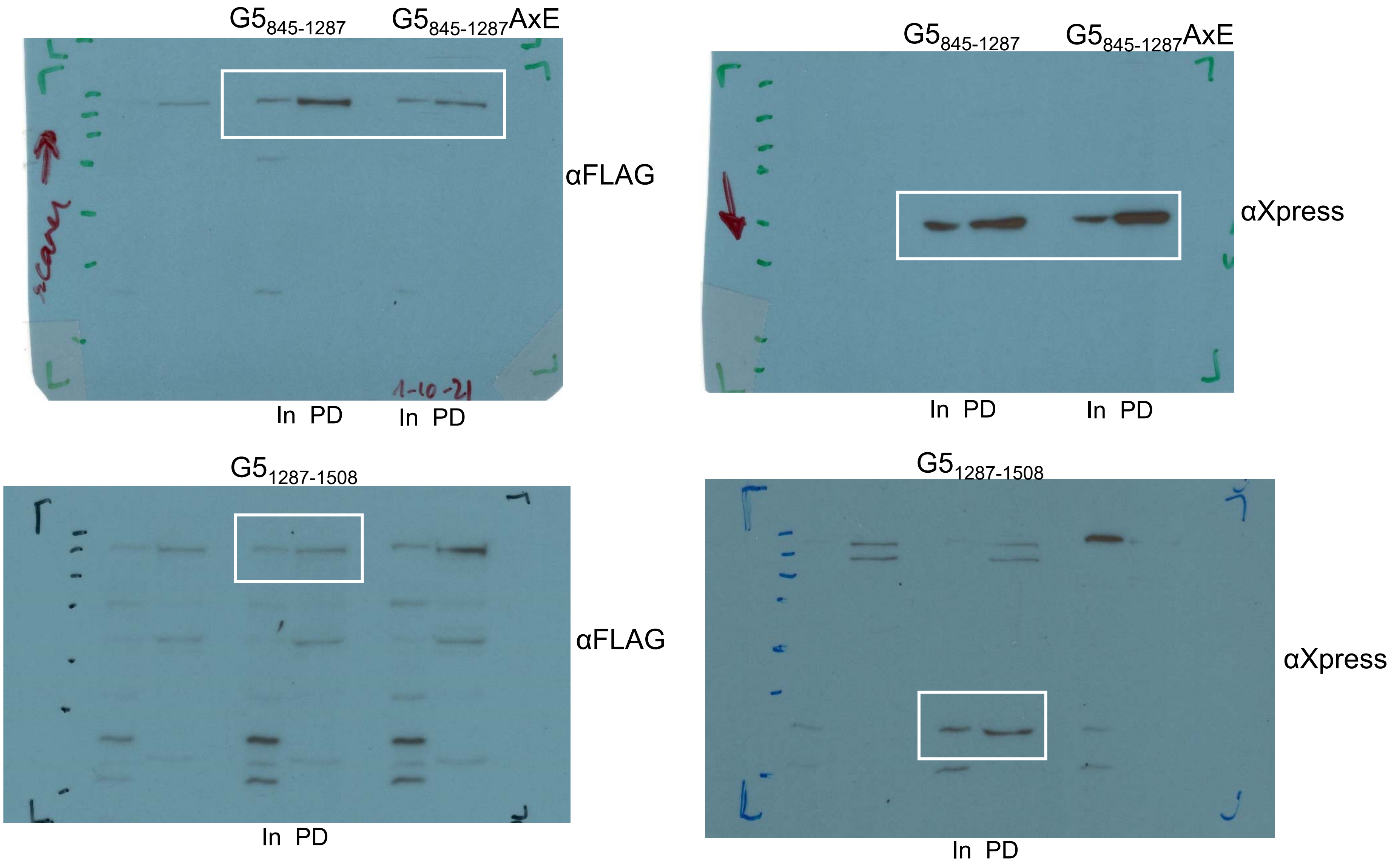

G5845-1287
G5845-1287AxE
αFLAG
In PD
In PD
G5845-1287
G5845-1287AxE
αXpress
In PD
In PD
G51287-1508
αFLAG
In PD
G51287-1508
αXpress
In PD

Fig. 3B

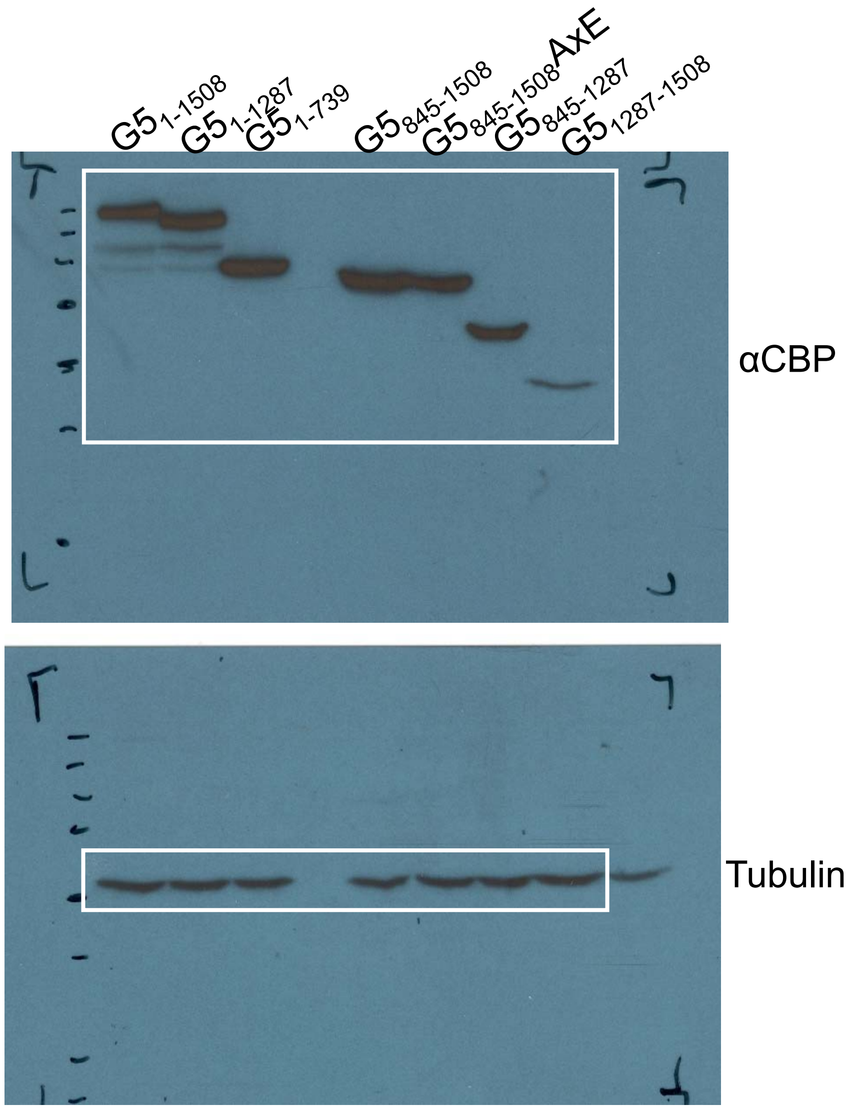
G5 1-1508
G5 1-1287
G5 1-739
G5 845-1508
G5 845-1508 AxE
G5 845-1287
G5 1287-1508
αCBP
Tubulin

Fig. 3C

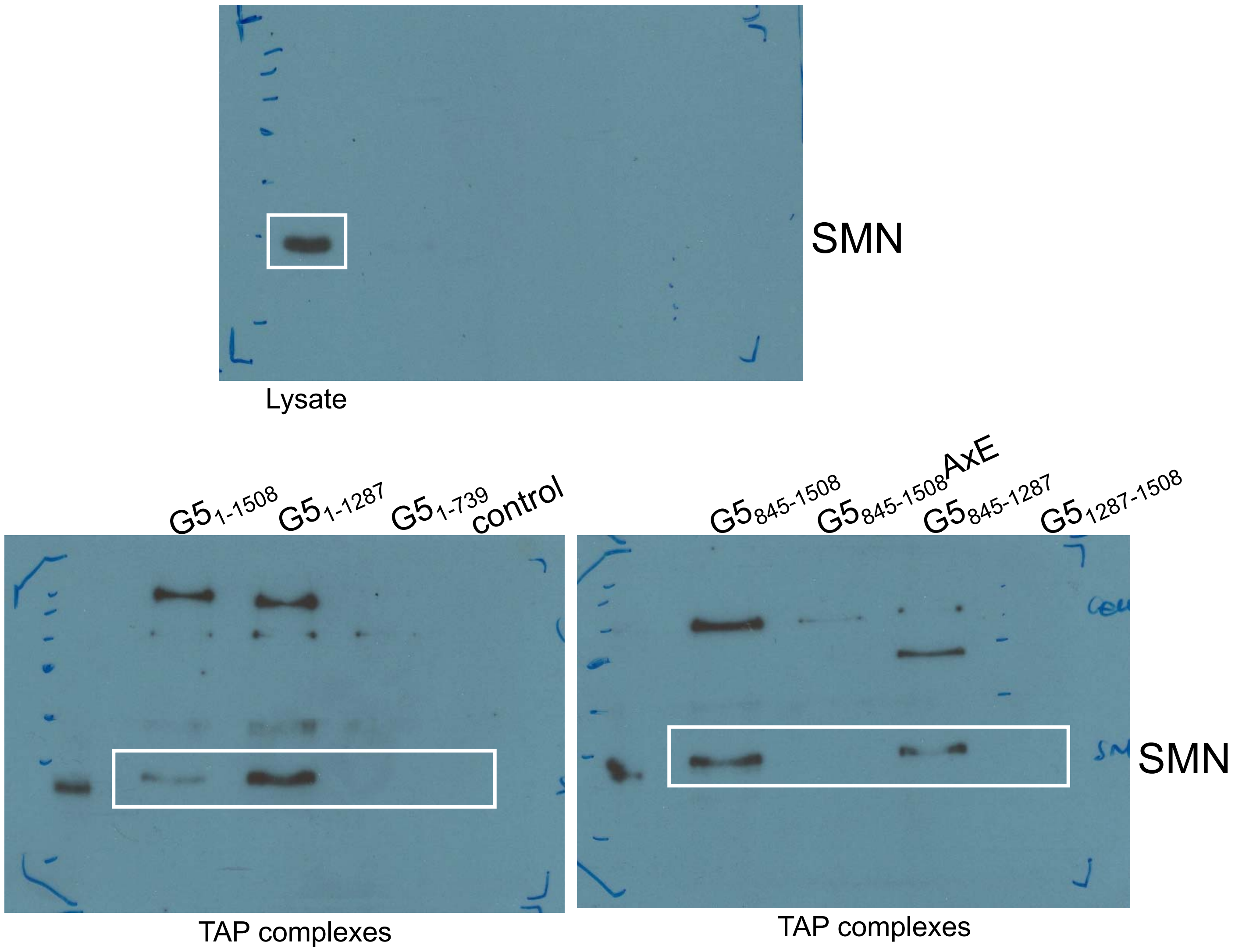

SMN
Lysate
G5 1-1508
G5 1-1287
G5 1-739
control
G5 845-1508
G5 845-1508 AxE
G5 845-1287
G5 1287-1508
SMN
TAP complexes
TAP complexes

Fig. 3C

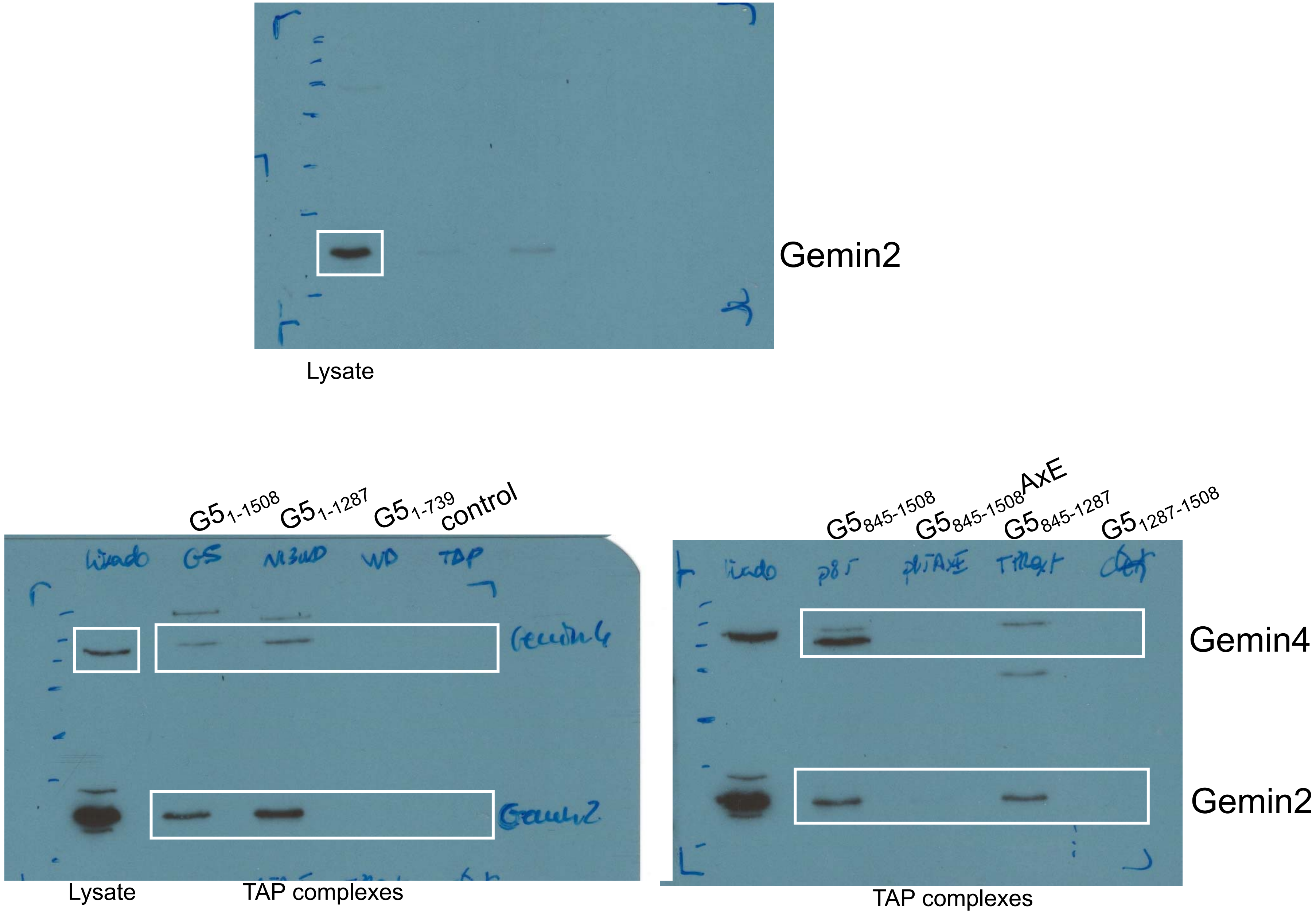
Gemin2
Lysate
G5$_{1-1508}$
G5$_{1-1287}$
G5$_{1-739}$
control
Lysate
TAP complexes
G5$_{845-1508}$
G5$_{845-1508}$AxE
G5$_{845-1287}$
G5$_{1287-1508}$
Gemin4
Gemin2
TAP complexes

Fig. 5B

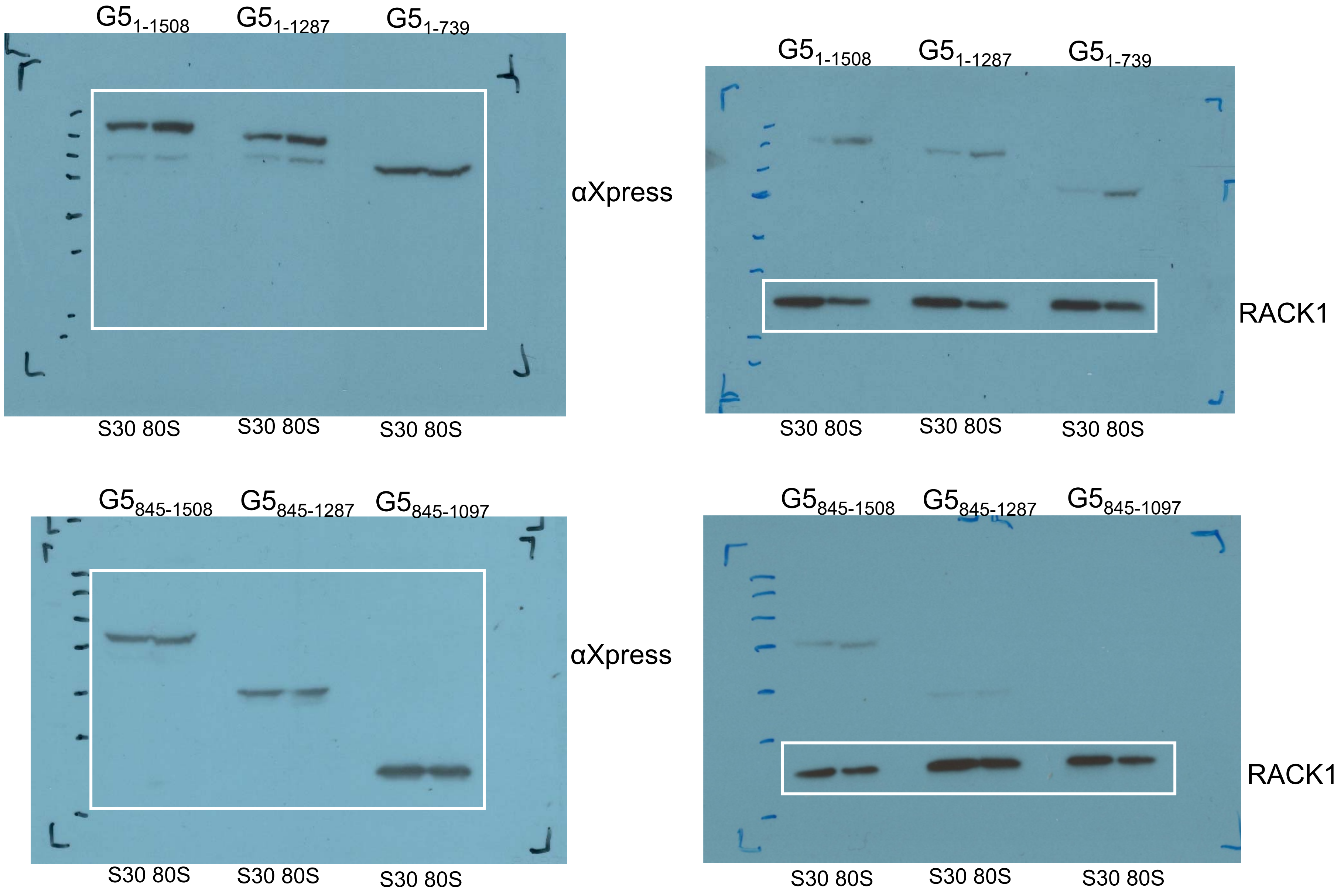
$G5_{1-1508}$ $G5_{1-1287}$ $G5_{1-739}$
αXpress
S30 80S S30 80S S30 80S
$G5_{1-1508}$ $G5_{1-1287}$ $G5_{1-739}$
RACK1
S30 80S S30 80S S30 80S
$G5_{845-1508}$ $G5_{845-1287}$ $G5_{845-1097}$
αXpress
S30 80S S30 80S S30 80S
$G5_{845-1508}$ $G5_{845-1287}$ $G5_{845-1097}$
RACK1
S30 80S S30 80S S30 80S

Fig. 5B

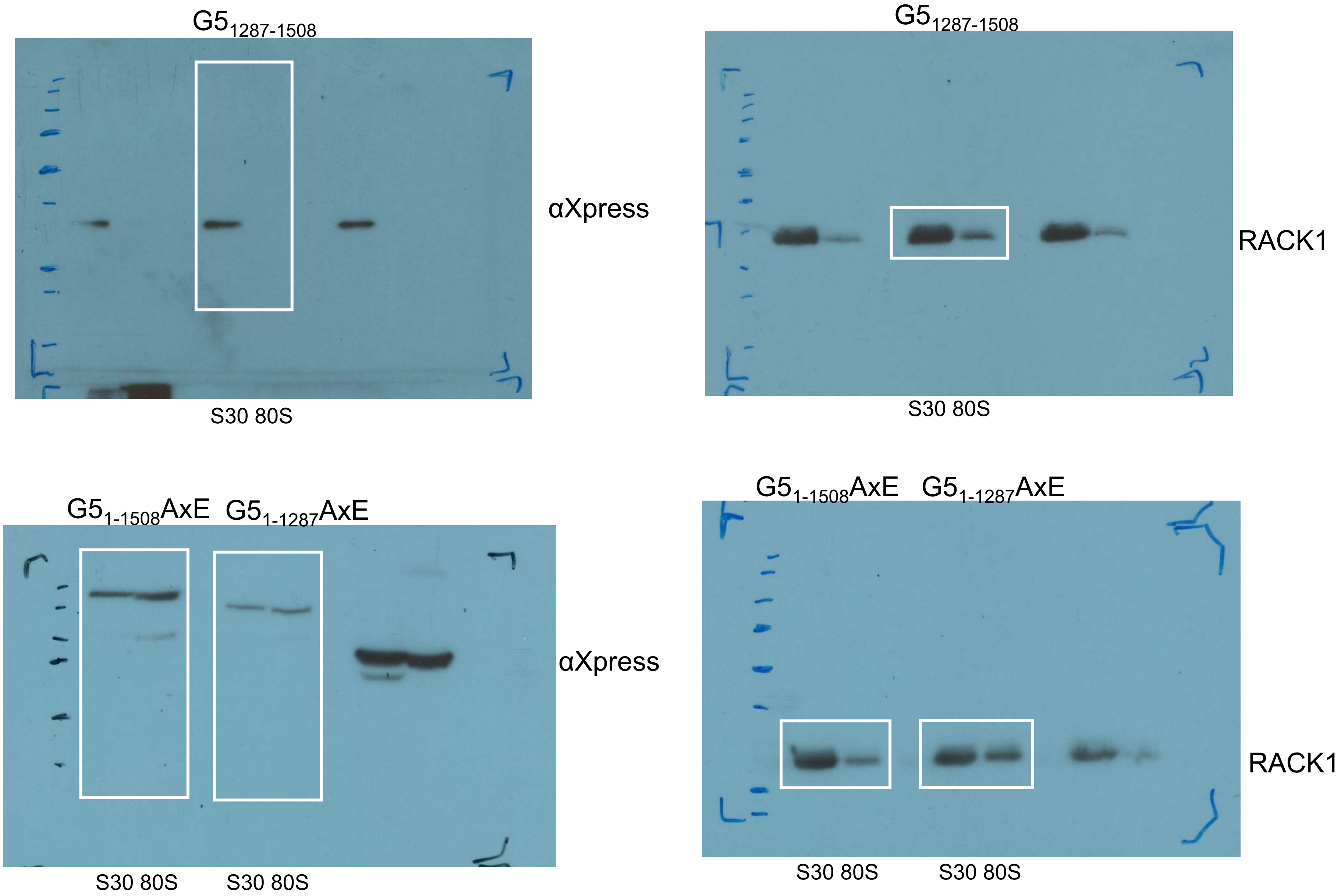
G5 1287-1508
αXpress
S30 80S
G5 1287-1508
RACK1
S30 80S
G5 1-1508 AxE
G5 1-1287 AxE
αXpress
S30 80S
S30 80S
G5 1-1508 AxE
G5 1-1287 AxE
RACK1
S30 80S
S30 80S

Fig. 5B

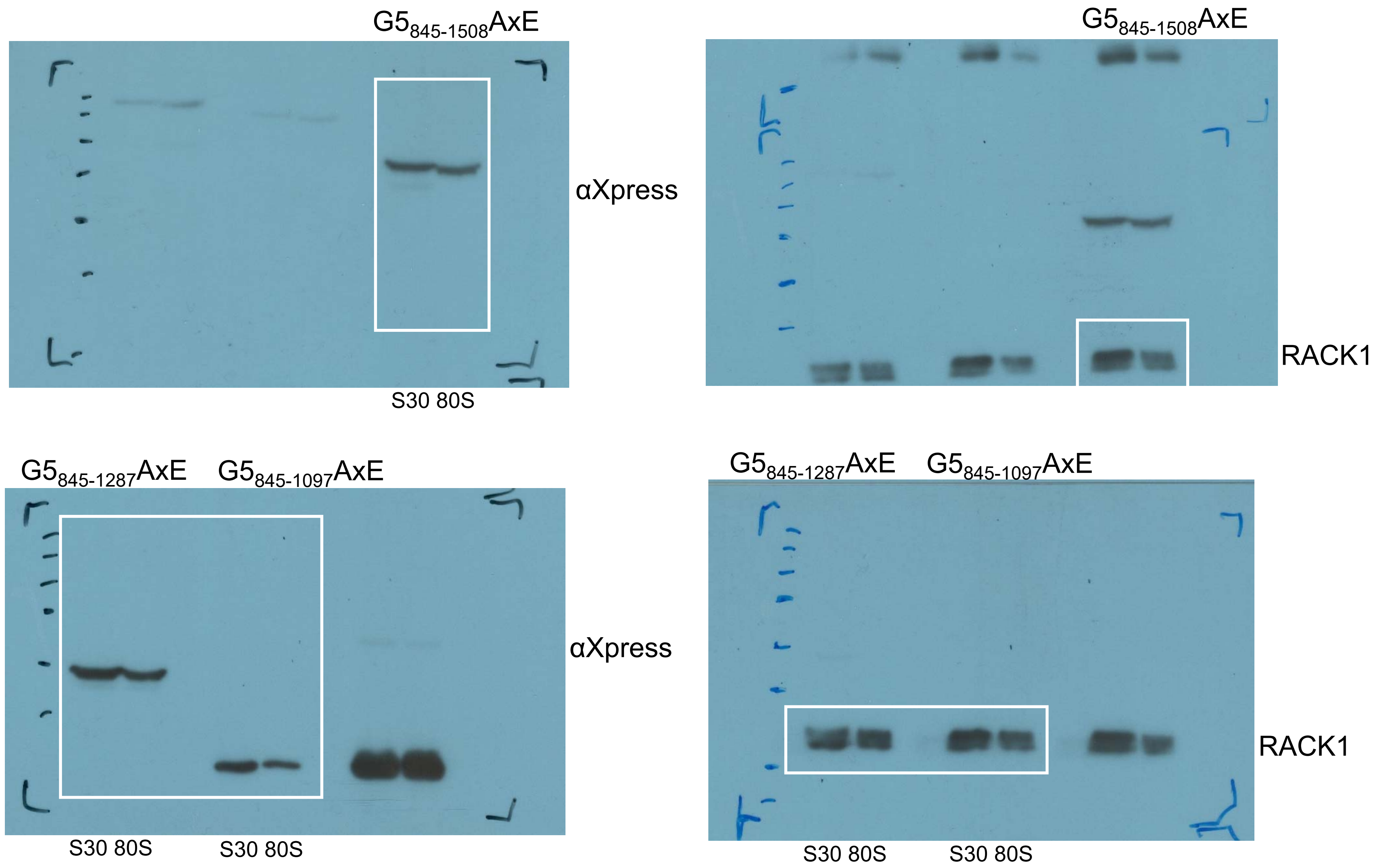

G5845-1508AxE
αXpress
S30 80S
G5845-1508AxE
RACK1
G5845-1287AxE
G5845-1097AxE
αXpress
S30 80S
S30 80S
G5845-1287AxE
G5845-1097AxE
RACK1
S30 80S
S30 80S

Fig. 5F

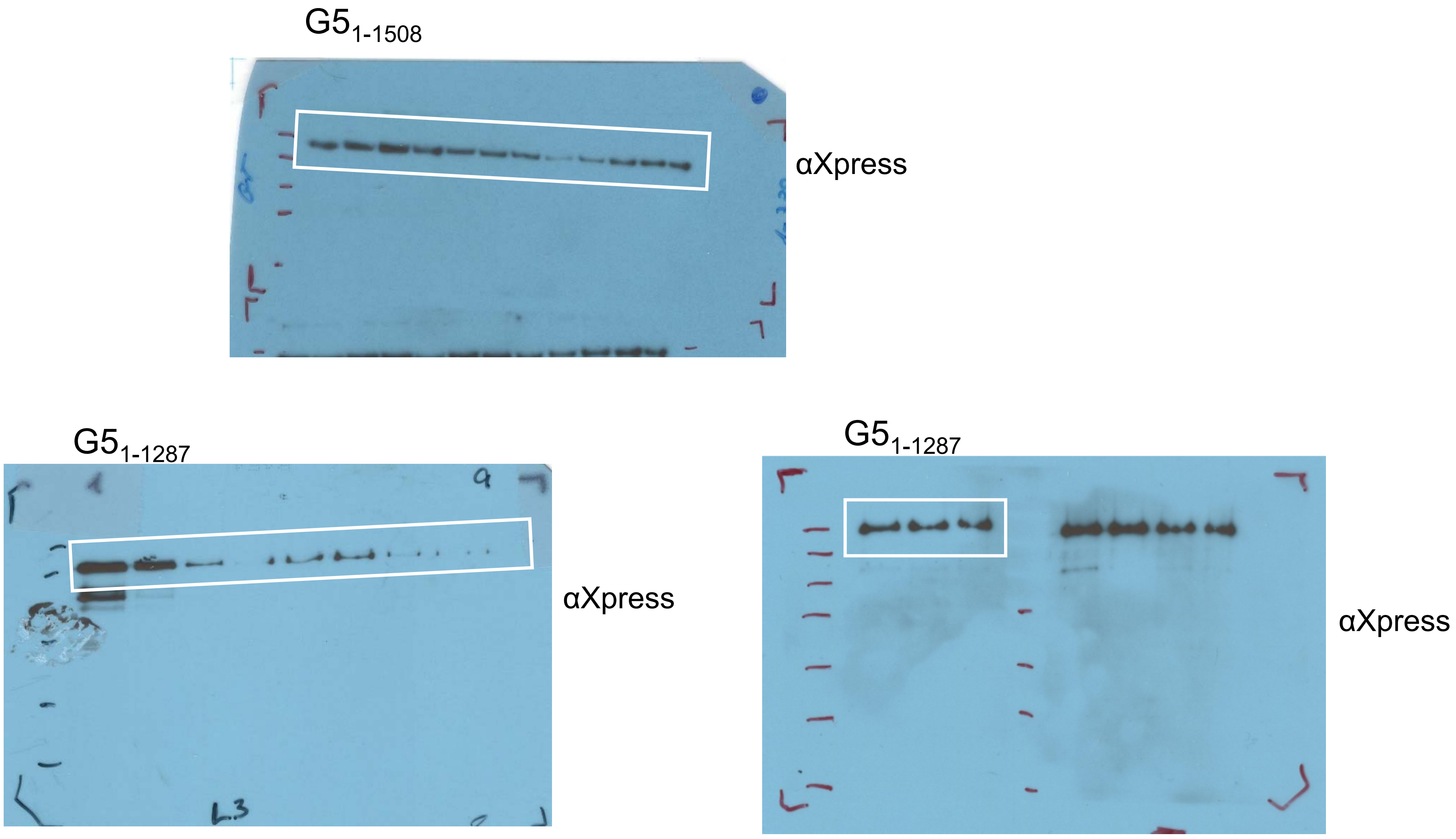

Fig. 5F

$G5_{1-739}$

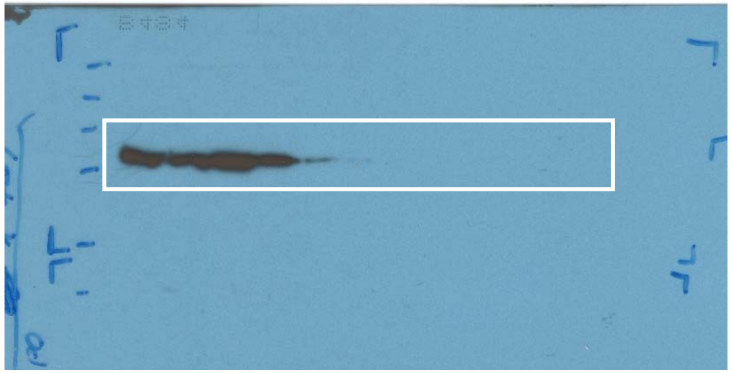

αXpress

$G5_{845-1508}$

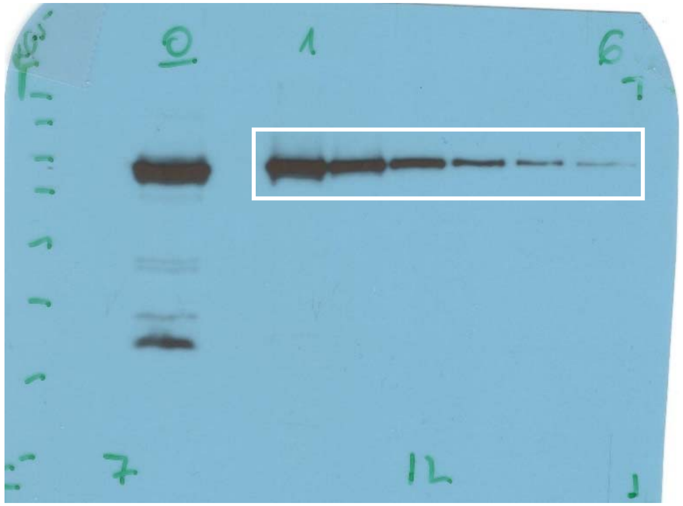

αXpress

$G5_{845-1508}$

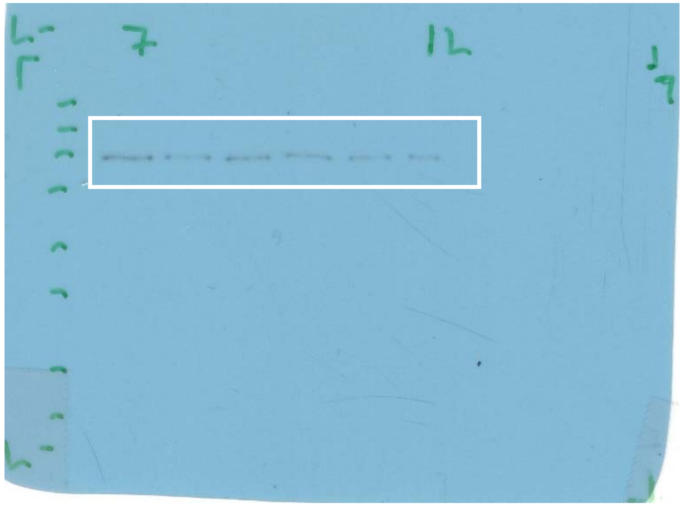

αXpress

Fig. 5F

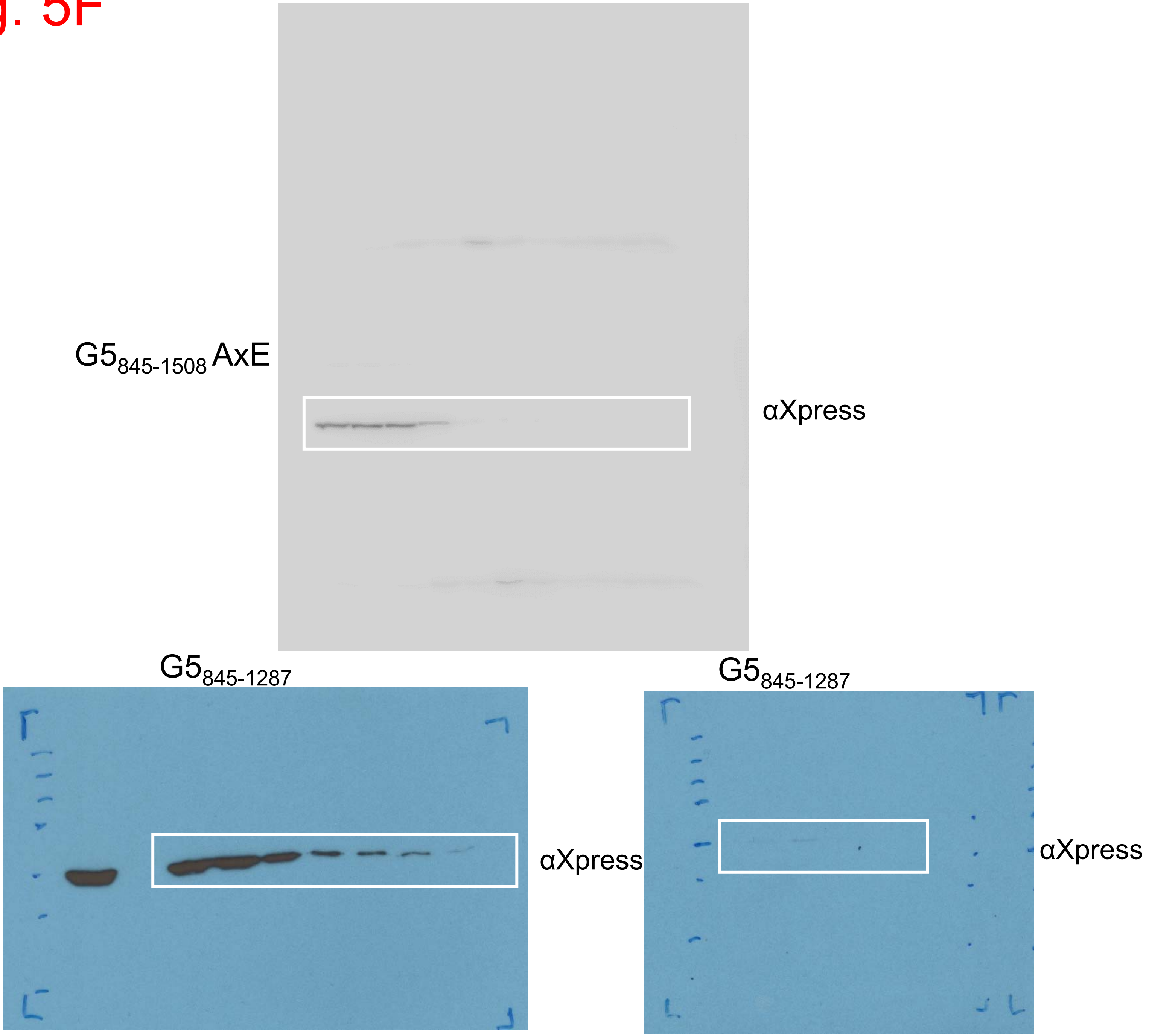
G5845-1508 AxE
αXpress
G5845-1287
αXpress
G5845-1287
αXpress

Fig. 5F

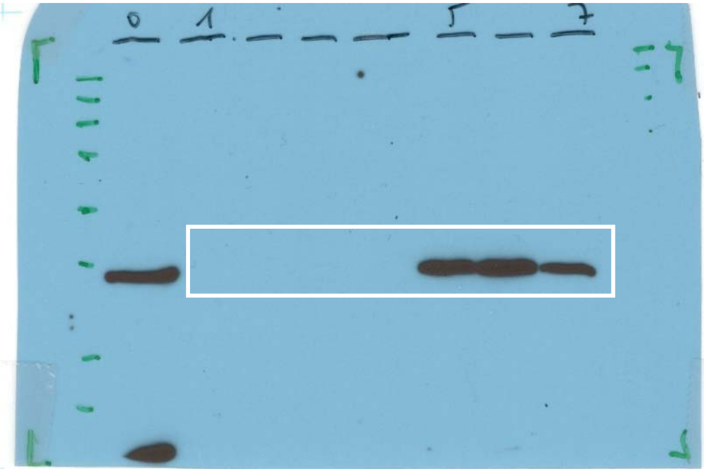

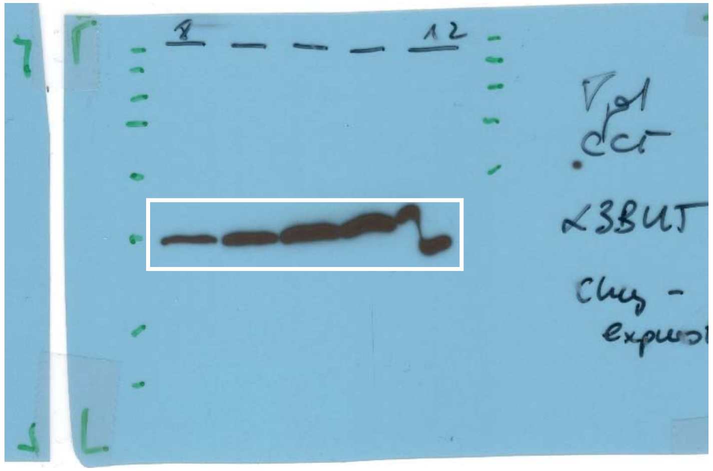

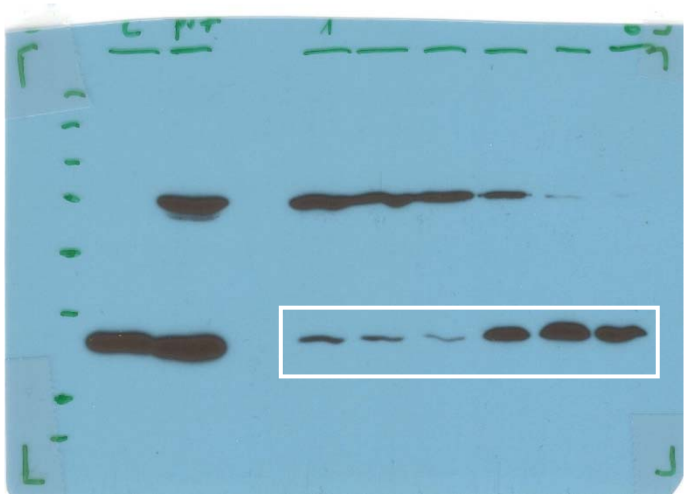

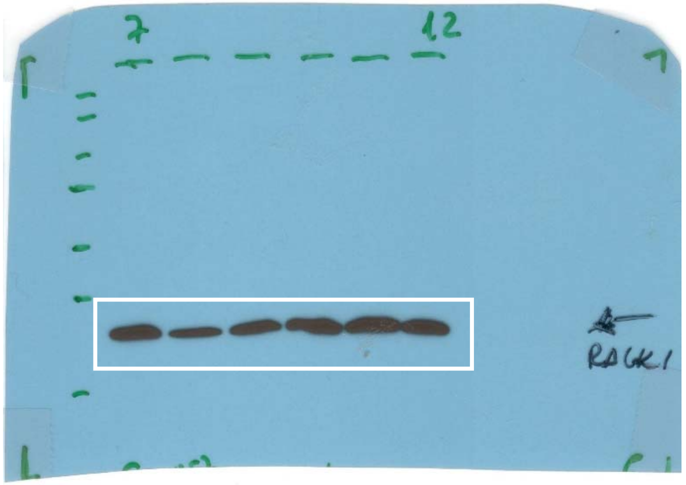

Supplementary Fig. S1

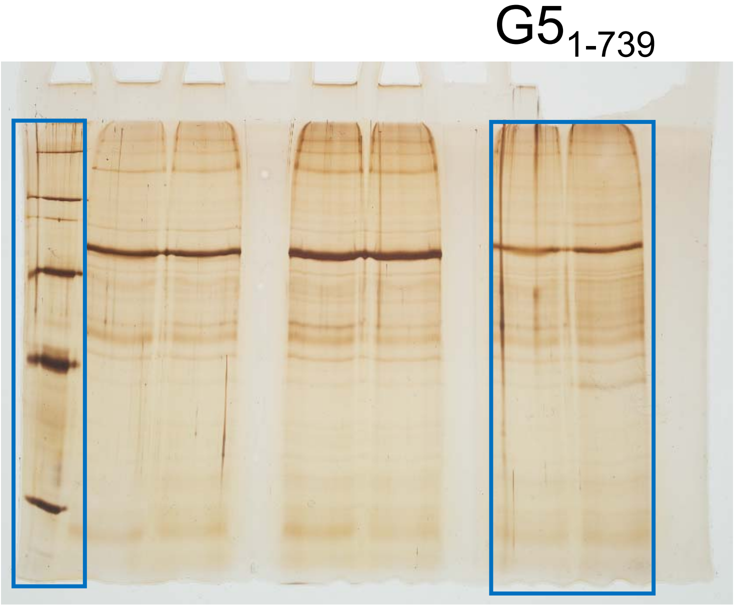

Supplementary Fig. S2

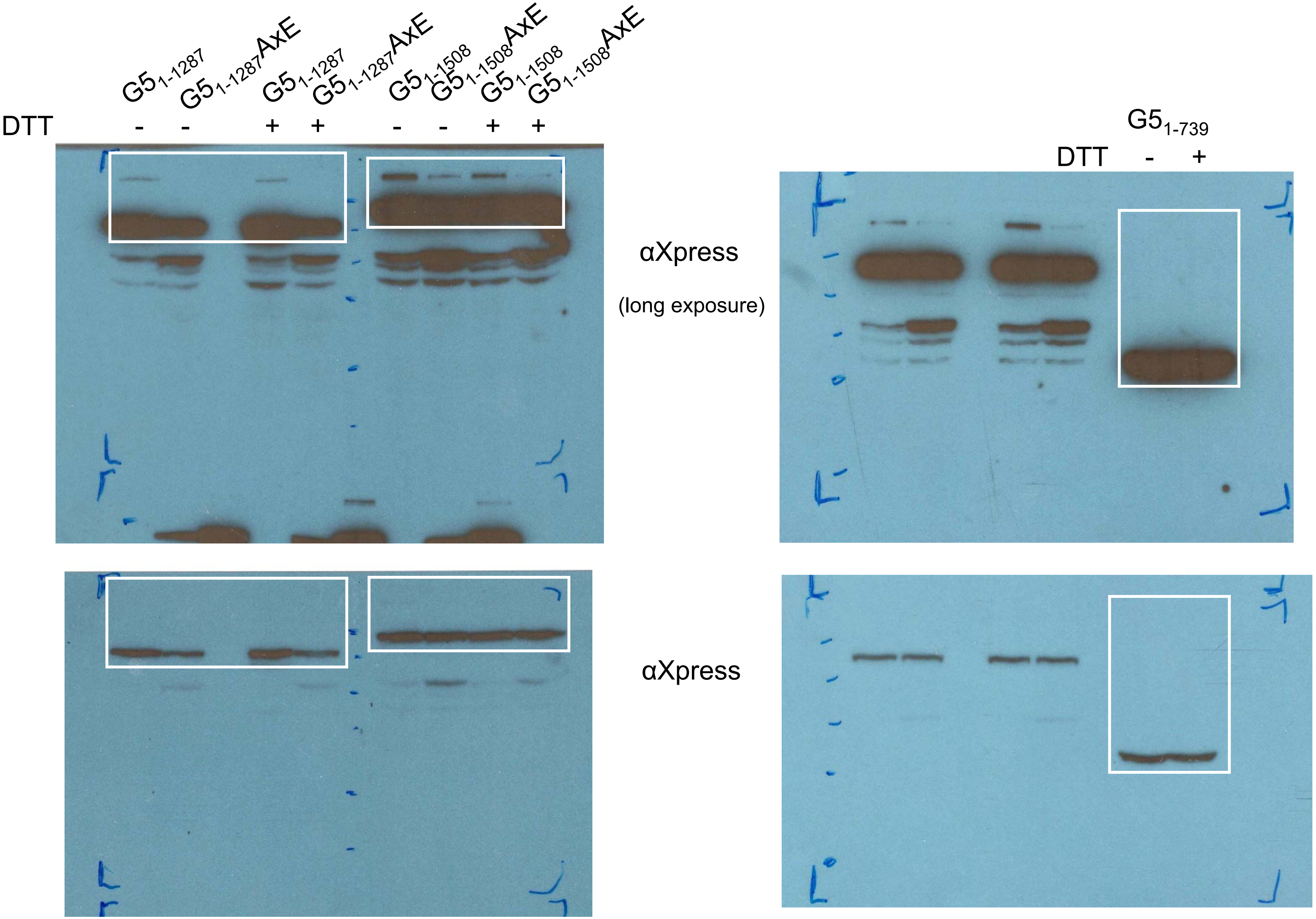

Supplementary Fig. S2

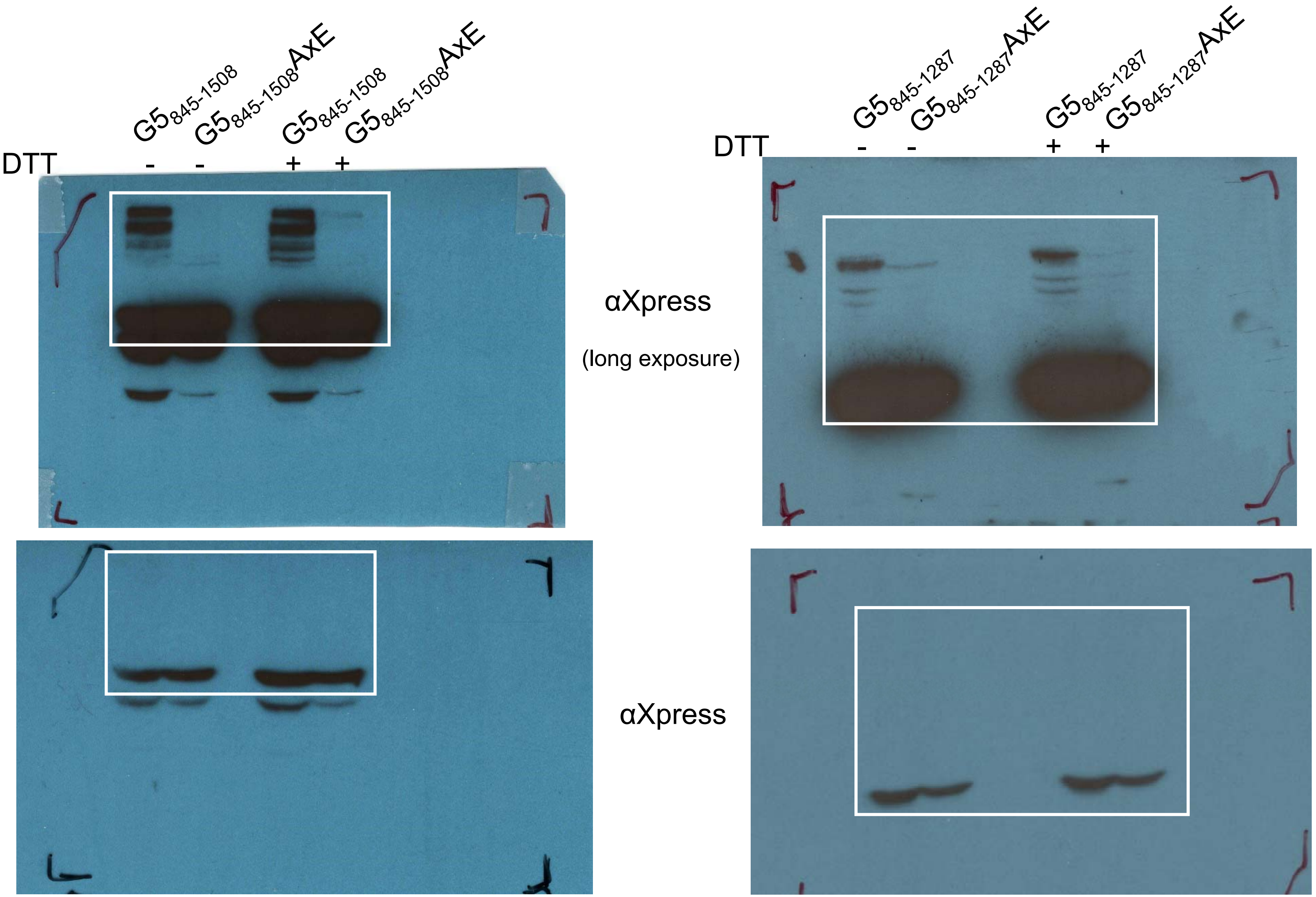

Supplement: Supplementary file 2 — Original data files [file 41420_2024_2057_MOESM2_ESM.pdf]
