# Supplementary material for: Oligomerization regulates the interaction of Gemin5 with members of the SMN complex and the translation machinery
Source: Cell Death Discov. 2024 Jun 28;10:306. doi: 10.1038/s41420-024-02057-5 (PMC11213948; doi:10.1038/s41420-024-02057-5)
Supplement: Supplementary file 3 — Supplementary material [file 41420_2024_2057_MOESM3_ESM.pdf]

Author contribution

R.F-V and E.M-S Conceptualization, methodology; R.F-V, S.A, and A.E-B Investigation, Validation, Software; E.M-S Original draft preparation, reviewing and editing.
